# Immune Checkpoint Inhibitors Combined With Chemotherapy Compared With Chemotherapy Alone for Triple-Negative Breast Cancer: A Systematic Review and Meta-Analysis

**DOI:** 10.3389/fonc.2021.795650

**Published:** 2021-12-16

**Authors:** Qiao Ji, Jingxian Ding, Meiqi Hao, Nachuan Luo, Jiabing Huang, Wenxiong Zhang

**Affiliations:** ^1^ Department of Radiation Oncology, The Third Hospital of Nanchang, Nanchang, China; ^2^ Department of Breast surgery, The Second Affiliated Hospital of Nanchang University, Nanchang, China; ^3^ Jiangxi Medical College, Nanchang University, Nanchang, China; ^4^ Department of Oncology, The Second Affiliated Hospital of Nanchang University, Nanchang, China; ^5^ Department of Cardiovascular Medicine, The Second Affiliated Hospital of Nanchang University, Nanchang, China; ^6^ Department of Thoracic Surgery, The Second Affiliated Hospital of Nanchang University, Nanchang, China

**Keywords:** chemotherapy, triple-negative breast cancer, meta-analysis, immune checkpoint inhibitors, systematic review

## Abstract

**Background:**

It is still controversial whether immune checkpoint inhibitors (ICIs) can improve the curative effect when added to original standard chemotherapy treatment for triple-negative breast cancer (TNBC). We compared their antitumor efficacy and adverse effects (AEs) to make a better clinical decision.

**Methods:**

Seven databases were searched for eligible articles. Progression-free survival (PFS), overall survival (OS), and AEs were measured as the primary outcomes.

**Results:**

Nine randomized controlled trials (RCTs) involving 4,501 patients were included. ICI+chemotherapy treatment achieved better PFS (hazard ratio [HR]: 0.78, [0.70–0.86], *p* < 0.00001), OS (HR: 0.86, [0.74–0.99], *p* = 0.04), and complete response (584/1,106 *vs.* 341/825, risk ratio [RR]: 1.38, [1.01–1.89], *p* = 0.04). With the prolongation of survival, the survival advantage of ICI+chemotherapy increased compared with chemotherapy. Subgroup analysis suggested that the addition of ICIs might not have a better effect in Asian patients, patients with locally advanced disease, or patients with brain metastases. In the toxicity analysis, more Grade 3–5 AEs and serious AEs were found in the ICI+chemotherapy group. For Grade 3–5 AEs, more cases of diarrhea, severe skin reactions, pneumonitis, hepatitis, and adrenal insufficiency were related to the ICI+chemotherapy group.

**Conclusions:**

ICI+chemotherapy appears to be better than chemotherapy alone for TNBC treatment, with better OS and PFS. However, its high rates of serious AEs need to be taken seriously.

**Systematic Review Registration:**

PROSPERO Registration: CRD42021276394.

## Introduction

In recent years, breast cancer has been the most common malignancy in women (1). As one of the major subtypes (15–20%), triple-negative breast cancer (TNBC) has the worst prognosis (2). In clinical practice, chemotherapy remains the standard of care (not only in neoadjuvant therapy but also in radical drug therapy) for patients with TNBC (3). However, its poor survival efficacy is not satisfactory for patients and doctors. In recent years, immune checkpoint inhibitors (ICIs) have been incorporated into cancer treatment, and their efficacy has been proven in lung cancer, hepatocellular carcinoma, and gastric cancer (4–6). However, whether ICIs can improve the curative effect when added to original standard chemotherapy treatment for TNBC is still controversial.

In the updated guidelines, ICIs+chemotherapy has been recognized as one of the treatment options for TNBC (7, 8). The KEYNOTE-522 and IMpassion130 trials compared ICIs+chemotherapy with chemotherapy in 2,076 patients with TNBC and suggested that combination therapy prolonged progression-free survival (PFS) and increased the rates of pathological complete response (PCR) (9, 10). Similar results were confirmed by 4 other randomized controlled trials (RCTs) (11–15). However, Bachelot et al.’s, Brufsky et al.’s, and Tolaney et al.’s studies reported that ICIs+chemotherapy could not improve the survival of patients but will cause more adverse effects (AEs) and reduce the quality of life of patients (16–18).

Hence, this meta-analysis of RCTs aimed to compare the efficacy and safety between ICIs+chemotherapy and chemotherapy for TNBC.

## Materials and Methods

We conducted this study according to the Preferred Reporting Items for Systematic Review and Meta-Analysis guidelines (PRISMA) (**Table S1**) (19). (PROSPERO Registration: CRD42021276394)?.

### Search Strategy

Studies were retrieved from the following databases: The Cochrane Library, PubMed, Web of Science, Scopus, EMBASE, ScienceDirect, and Ovid MEDLINE. Studies were retrieval time from inception to May 5, 2021. The MeSH terms and keywords were “Breast cancer”, “Immune checkpoint inhibitors”, and “Chemotherapy”. The search details are included in **Table S2**.

### Selection Criteria

The inclusion criteria were as follows: (1) RCTs published in English comparing ICIs+chemotherapy with chemotherapy alone; (2) studies that enrolled patients with TNBC; and (3) the outcomes included survival indicators (OS and PFS), drug responses, and AEs.

The exclusion criteria were as follows: (1) animal studies; (2) meta-analyses and reviews; (3) conference articles; (4) case reports; and (5) studies that did not only enroll patients with TNBC.

### Data Extraction

Two investigators extracted the following information independently: the publication year, first author, participant characteristics (quantity, age, etc.), tumor characteristics (histopathology, stage, etc.), antitumor efficacy (OS, PFS, etc.), and number of AEs. All disagreements were resolved by a third investigator.

### Outcome Assessments

PFS and OS were the primary outcomes. The subgroup analysis of OS was performed according to age, race, Eastern Cooperative Oncology Group (ECOG) performance status, baseline disease status, metastatic sites, PD-L1 status, neoadjuvant therapy, homologous recombination deficiency (HRD), metastases (brain, bone, liver, or lung), lymph node-only disease, and previous treatment (chemotherapy, taxane, or anthracycline).

### Quality Assessment

We assessed the quality of the included RCTs by using the Cochrane Risk of Bias Tool (CRBT) (20) and 5-point Jadad scale (21). We assessed the quality of the results by using the Grading of Recommendations Assessment, Development and Evaluation (GRADE) system (22).

### Statistical Analysis

When analyzing survival outcomes (PFS, OS, etc.), we used hazard ratios (HRs). When analyzing dichotomous variables (PFSR, OSR, complete response [CR], AEs, etc.), we used risk ratios (RRs). Heterogeneity was evaluated by the *I^2^* statistic and χ^2^ test. A random-effects model was used when heterogeneity was significant (*I^2^* < 50% or *p* > 0.1); otherwise, a fixed-effects model was used. Publication bias was evaluated through visual inspection of the funnel plots. A *P* < 0.05 was identified as statistically significant. All analyses were performed using Review Manager 5.3 and SPSS 18.0.

## Results

### Search Results

**Figure 1:**
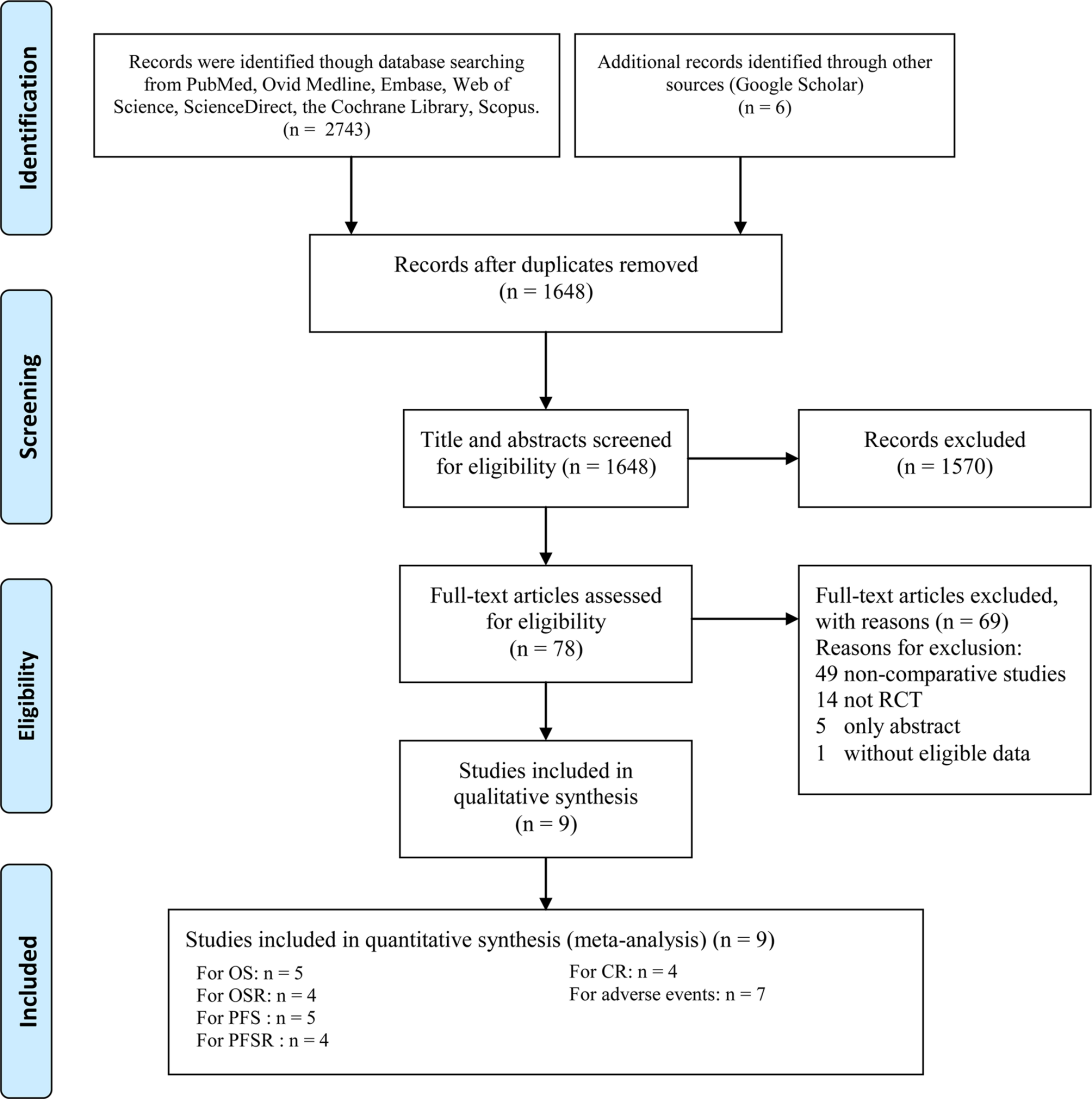
Flow chart of study selection.

Nine RCTs involving 4,501 patients (2,645 patients in the ICI+chemotherapy group and 1,856 patients in the chemotherapy group) were included (9–16, 18) (**Figure 1**). Two studies (14, 16) were conducted in Europe, one (18) was conducted in the USA, and the other five studies were global studies (9–13). The essential information of the included studies is summarized in **Table 1**. According to the Jadad scale (**Table S3**) and CRBT (**Figure S1**), all eight RCTs were of high quality. According to the GRADE system, the evidence grades of all the results were medium-high.

**Table 1:** Characteristics of the included randomized controlled trials.

Study				Period	Groups	Patients (n)	Median age (year)	Stage	PD-L1+	Treatment	Follow-upduration,mo	Design
2021	Miles et al. (15)	IMpassion131	Phase III	2017.08–2019.09	ICIs+Chemotherapy	431	54	Stage IV	191	Atezolizumab, 840mg (d1, 15) + Paclitaxel, 90 mg/m² (d1, 8, 15), q4w until PD	8.8	RCT
					Chemotherapy	220	53		101	Paclitaxel, 90 mg/m² (d1, 8, 15), q4w until PD	8.5	
2021	Bachelot et al. (16)	SAFIR02-BREAST IMMUNO	Phase II	2016.01–2019.09	ICIs+Chemotherapy	47	56	Stage IV	10	Durvalumab, 10 mg/kg, q2w+Chemotherapy (doctor’s choice), until PD	19.7	RCT
					Chemotherapy	35	56.5		8	Chemotherapy (doctor’s choice), until PD		
2020	Schmid et al. (9)	KEYNOTE-522	Phase III	2017.03–2018.09	ICIs+Chemotherapy	784	49	Stage II–III	656	Pembrolizumab, 200 mg, q3w+Paclitaxel, 80 mg/m², q1w+carboplatin^a^, for 12w (first neoadjuvant treatment); followed by Pembrolizumab, 200 mg, q3w+Doxorubicin, 60 mg/m², q3w (or Epirubicin, 90 mg/m², q3w) +cyclophosphamide, 600 mg/m², q3w, for 12w (second neoadjuvant treatment). After definitive surgery, pembrolizumab, 200 mg, q3w for up to 9 cycles.	15.5	RCT
					Chemotherapy	390	48		317	Placebo, q3w+Paclitaxel, 80 mg/m², q1w+carboplatin^a^, for 12w (first neoadjuvant treatment); followed by Placebo, q3w+Doxorubicin, 60 mg/m², q3w (or Epirubicin, 90 mg/m², q3w) +cyclophosphamide, 600 mg/m², q3w, for 12w (second neoadjuvant treatment); after definitive surgery, placebo, q3w for up to 9 cycles.		
2020	Schmid et al. (10)	IMpassion130	Phase III	2015.06–2017.05	ICIs+Chemotherapy	451	55	Stage IV	185	Atezolizumab, 840 mg (d1, 15)+Nab-paclitaxel, 100 mg/m² (d1, 8, 15), q4w until PD	18.5	RCT
					Chemotherapy	451	56		184	Nab-paclitaxel, 100 mg/m² (d1, 8, 15), q4w until PD	17.5	
2020	Mittendorf et al. (11)	IMpassion031	Phase III	2017.07–2019.09	ICIs+Chemotherapy	165	51	Stage II–III	78	Atezolizumab,840 mg, q2w+Nab-paclitaxel, 125 mg/m², qw, for 12 weeks, followed by Atezolizumab,840 mg, q2w+Doxorubicin, 60 mg/m²+Cyclophosphamide, 600 mg/m², q2w for 8w; after surgery, atezolizumab,1,200 mg, q3w for 11 cycles	20.6	RCT
					Chemotherapy	168	51		76	Nab-paclitaxel, 125 mg/m², qw, for 12 weeks, followed by Doxorubicin, 60 mg/m²+ Cyclophosphamide, 600 mg/m², q2w for 8w; after surgery, subsequently monitored for 1 year	19.8	
2020	Cortes et al. (12)	KEYNOTE-355	Phase III	2017.01–2018.06	ICIs+Chemotherapy	566	53	Stage IV	425	Pembrolizumab, 200 mg q3w+(Nab-paclitaxel, 100 mg/m², d1, 8, 15, q4w or Paclitaxel, 90 mg/m², d1, 8, 15, q4w or Gemcitabine, 1,000 mg/m²+Carboplatin, d1, 8, q3w) until PD	25.9	RCT
					Chemotherapy	281	53		211	Nab-paclitaxel, 100 mg/m², d1, 8, 15, q4w or Paclitaxel, 90 mg/m², d1, 8, 15, q4w or Gemcitabine, 1,000 mg/m²+Carboplatin, d1, 8, q3w until PD	26.3	
2020	Nanda et al. (13)	I-SPY2	Phase II	2015.11–2016.11	ICIs+Chemotherapy	69	50	Stage I–III	–	Pembrolizumab, 200 mg, q3w+Paclitaxel, 80 mg/m2, d1, 7, 14+Doxorubicin, 60 mg/m², d1, 14+Cyclophosphamide, 600 mg/m2, d1,14 for 4 cycles	33.6	RCT
					Chemotherapy	181	47		–	Paclitaxel, 80 mg/m2, d1, 7, 14+Doxorubicin, 60 mg/m2, d1, 14+Cyclophosphamide, 600 mg/m2, d1, 14 for 4 cycles		
2020	Tolaney et al. (18)	–	Phase II	2017.04–2018.08	ICIs+Chemotherapy	44	58	Stage IV	–	Pembrolizumab, 200 mg, q3w+Eribulin,1.4 mg/m², d1, 8, q3w until PD	10.5	RCT
					Chemotherapy	44	57		–	Eribulin, 1.4 mg/m2, d1, 8, q3w until PD		
2019	Loibl et al. (14)	GeparNuevo	Phase II	2016.06–2017.10	ICIs+Chemotherapy	88	49.5	Stage I–III	69	One injection durvalumab, 0.75 g 2w followed by durvalumab 1.5 g, q4w+Nabpaclitaxel, 125 mg/m², q1w for 12w, followed by Durvalumab, 1.5 g, q4w +dose-dense Epirubicin/Cyclophosphamide, q2w for 4 cycles.	–	RCT
					Chemotherapy	86	49.5		69	One injection placebo, 2w followed by placebo, q4w+Nabpaclitaxel, 125 mg/m², q1w for 12w, followed by placebo, q4w+dose-dense Epirubicin/Cyclophosphamide, q2w for 4 cycles.		

RCT, randomized controlled trial; NSCLC, non-small-cell lung cancer; PD-L1+, programmed death ligand 1 positive; PD, progressive disease; ICIs, immune checkpoint inhibitors.

a At a dose based on an area under the concentration-time curve of 5 mg per milliliter per minute once every 3 weeks or 1.5 mg per milliliter per minute once weekly in the first 12 weeks.

### Antitumor Efficacy

**Figure 2:**
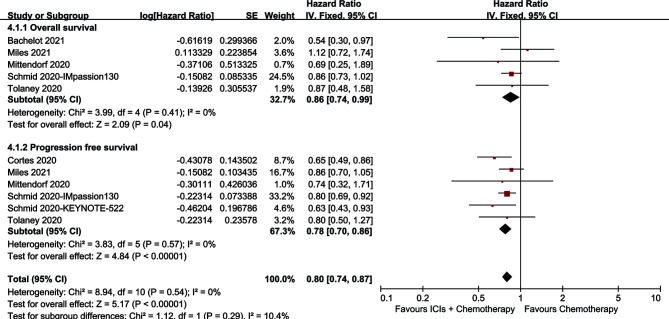
Forest plots of OS and PFS associated with ICIs+Chemotherapy *versus* Chemotherapy.

**Figure 3:**
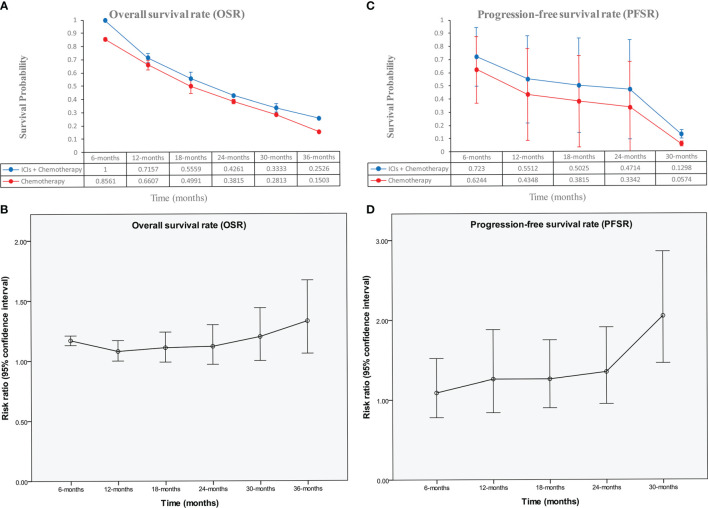
Comparisons of OSR (6–36 months, **A, B**), and PFSR (6–30 months, **C, D**) associated with ICIs+Chemotherapy *versus* Chemotherapy according to survival time.

The ICI+chemotherapy group achieved better OS than the chemotherapy group (HR: 0.86, [0.74–0.99], *p* = 0.04; **Figure 2**). At all points in time, the overall survival rate (OSR) tended to favor the ICI+chemotherapy group (OSR-6 m, RR: 1.17, [1.13–1.21], *p* < 0.00001; OSR-12 m, RR: 1.08, [1.00–1.17], *p* = 0.05; OSR-18 m, RR: 1.11, [0.99–1.24], *p* = 0.07; OSR-24 m, RR: 1.12, [0.97–1.30], *p* = 0.13; OSR-30 m, RR: 1.20, [1.00–1.44], *p* = 0.04; OSR-36 m, RR: 1.33, [1.06–1.67], *p* = 0.01, **Figure S2**). With prolonged survival time, ICI+chemotherapy had an increasing advantage for OS (**Figures 3A, B**).

The ICI+chemotherapy group achieved better PFS than the chemotherapy group (HR: 0.78, [0.70–0.86], *p* < 0.00001; **Figure 2**). At all points in time, the progression-free survival rate (PFSR) significantly favored the ICIs+Chemotherapy group (PFSR-6 m, RR: 1.09, [0.78–0.1.52], *p* = 62; PFSR-12 m, RR: 1.26, [0.84–1.88], *p* = 0.27; PFSR-18 m, RR: 1.26, [0.90–1.75], *p* = 0.18; PFSR-24 m, RR: 1.35, [0.95–1.91], *p* = 0.10; PFSR-30 m, RR: 0.2.05, [1.46–2.86], *p* < 0.0001, **Figure S3**). With prolonged survival time, ICI+chemotherapy had an increasing advantage for PFS (**Figures 3C, D**).

In the subgroup analysis, the favorable tendency of OS did not show significant changes according to age, ECOG performance status, number of metastatic sites, PD-L1 status, neoadjuvant therapy, lymph node-only disease, bone metastases, liver metastases, lung metastases, or previous chemotherapy (chemotherapy, taxane, or anthracycline). The addition of ICIs might have the opposite effect in the subgroups by race (Asian), baseline disease status (locally advanced), and brain metastases (yes) (**Table 2**).

**Table 2:** Subgroup analysis for OS.

Subgroups	Included studies	Total	ICIs+Chemotherapy		Chemotherapy		HR (95% CI)
			Events	n	Events	n	
**All patients**	5	2,056	651	1138	566	918	0.79 (0.63,0.99)
**Age**							
18–40 years	1	114	44	63	37	51	0.81 (0.52,1.25)
41–64 years	1	569	158	284	170	285	0.88 (0.71,1.10)
>65 years	1	219	53	104	72	115	0.78 (0.55,1.12)
**Race**							
White	1	609	180	308	198	301	0.80 (0.65,0.98)
Asian	1	161	39	85	34	76	1.17 (0.74,1.87)
Black or African-American	1	58	14	26	21	32	0.75 (0.38,1.49)
**ECOG performance status**							
0	1	526	127	256	145	270	0.85 (0.67,1.08)
1	1	372	127	193	132	179	0.85 (0.66,1.08)
**Baseline disease status**							
Locally advanced	1	88	21	46	13	42	1.53 (0.76,3.06)
Metastatic	1	812	234	404	266	408	0.82 (0.90,0.98)
**Number of metastatic sites**							
0-3	1	673	172	332	194	341	0.83 (0.68,1.02)
4+	1	226	83	118	83	108	0.90 (0.66,1.22)
**PD-L1 status**							
PD-L1 positive	4	717	206	407	181	310	0.79 (0.63,0.99)
PD-L1 negative	2	562	175	283	179	279	0.56 (0.23,1.38)
**Neoadjuvant therapy**							
Yes	1	88	25	44	27	44	0.87 (0.48,1.58)
No	4	1,968	626	1,092	539	874	0.86 (0.74,0.99)
**Homologous recombination deficiency (HRD)**							
Low HRD	1	21	3	10	8	11	0.27 (0.07,1.10)
High HRD	1	31	9	19	9	12	0.71 (0.26,1.89)
**Brain metastases**							
Yes	1	61	22	30	19	31	1.34 (0.72,2.48)
No	1	841	233	421	260	420	0.83 (0.70,1.00)
**Bone metastases**							
Yes	1	286	92	145	103	141	0.80 (0.61,1.07)
No	1	616	163	306	176	310	0.88 (0.71,1.09)
**Liver metastases**							
Yes	1	244	88	126	95	118	0.77 (0.58,1.03)
No	1	658	167	325	184	333	0.88 (0.72,1.09)
**Lung metastases**							
Yes	1	469	138	227	153	242	0.94 (0.74,1.18)
No	1	433	117	224	126	209	0.80 (0.62,1.02)
**Lymph node-only disease**							
Yes	1	56	12	33	11	23	0.74 (0.32,1.67)
No	1	843	243	417	266	426	0.88 (0.74,1.05)
**Previous neoadjuvant or adjuvant chemotherapy**							
Yes	1	570	160	284	166	286	0.92 (0.74,1.15)
No	1	332	95	167	113	165	0.75 (0.57,0.99)
**Previous taxane treatment**							
Yes	1	461	138	231	136	230	0.95 (0.75,1.20)
No	1	441	117	220	143	221	0.76 (0.59,0.97)
**Previous anthracycline treatment**							
Yes	1	485	143	243	144	242	1.00 (0.79,1.26)
No	1	417	112	208	135	209	0.71 (0.55,0.92)

PD-L1+, programmed death ligand 1 positive; ICIs, immune checkpoint inhibitors; HR, hazard ratio; CI, confidence interval; OS, overall survival; HRD, homologous recombination deficiency; ECOG, Eastern Cooperative Oncology Group.

**Figure 4:**
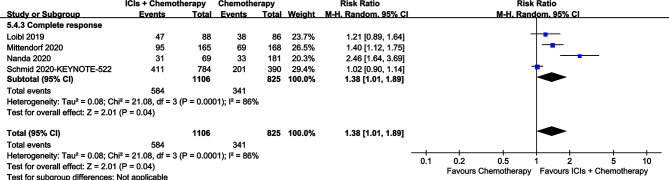
Forest plots of CR associated with ICIs+Chemotherapy *versus* Chemotherapy.

The CR of the ICI+chemotherapy group was higher than that of the chemotherapy group (584/1,106 *vs.* 341/825, RR: 1.38, [1.01–1.89], *p* = 0.04; **Figure 4**).

### Toxicity

In summary, ICI+chemotherapy treatment was related to more Grade 3–5 AEs, treatment-related Grade 3–5 AEs, serious AEs, treatment-related serious AEs, and AEs leading to treatment discontinuation. Total AEs, treatment-related AEs, death, treatment-related death, and AEs leading to dose reduction/dose interruption were comparable between the two groups (**Table 3**).

**Table 3:** Summary of adverse events.

Adverse events	Studies involved	ICIs+Chemotherapy			Chemotherapy		Risk ratio	95% CI	*I* ^2^(%)	P
		Event/total	%		Event/total	%				
**Total adverse events**	7	2462/2488	95.95%		1550/1589	97.55%	1.01	0.99-1.03	85	0.41
**Treatment-related adverse events**	5	1951/2013	96.92%		1255/1325	94.72%	1.02	0.98-1.06	88	0.43
**Grade 3-5 adverse events**	6	1697/2444	69.43%		901/1545	58.32%	1.14	1.03-1.25	69	0.0006
**Treatment-related grade 3-5 adverse events**	6	1295/2057	62.96%		724/1369	52.89%	1.09	1.03-1.16	45	0.002
**Serious adverse events**	2	155/616	25.16%		111/619	17.93%	1.40	1.13-1.74	19	0.002
**Treatment-related serious adverse events**	4	128/751	17.04%		88/740	11.89%	1.44	1.13-1.85	30	0.003
**Adverse event leading to treatment discontinuation**	4	123/751	16.38%		76/740	10.27%	1.61	1.24-2.10	46	0.0004
**Adverse event leading to dose reduction/dose interruption**	1	194/451	43.02%		173/451	38.36%	1.12	0.96-1.31	–	0.16
**Death**	3	7/663	1.06%		4/654	0.61%	1.76	0.52-5.97	0	0.37
**Treatment-related death**	1	2/451	0.44%		1/451	0.22%	2.00	0.18-21.98	–	0.57

ICIs, immune checkpoint inhibitors; CI, confidence interval.

For total AEs, increases in aspartate aminotransferase (AST) levels, vomiting, cough, rash, pyrexia, pruritus, infusion reaction, hypothyroidism, nail disorders, hypokalemia, hyperthyroidism, pneumonitis, hepatitis, and adrenal insufficiencies were related to the ICI+chemotherapy group. Total AEs greater than 10% are summarized in **Table 4**.

**Table 4:** Total adverse events an incidence of more than 10% according to combination of two groups.

Adverse events	Studies involved	ICIs+Chemotherapy		Chemotherapy		Total incidence	Risk ratio	95% CI	*I*2 (%)	P
		Event/total	%	Event/total	%				
Alopecia	5	1123/2054	54.67%	771/1376	56.03%	55.22%	1.03	0.97-1.09	0	0.33
Nausea	6	1135/2123	53.46%	825/1557	52.99%	53.26%	1.04	0.98-1.10	42	0.23
Infection	1	50/88	56.82%	39/86	45.35%	51.15%	1.25	0.93-1.68	–	0.13
Anemia	6	1004/2123	47.29%	640/1557	41.10%	44.67%	1.05	0.98-1.13	21	0.18
Fatigue	6	882/2123	41.54%	709/1557	45.54%	43.23%	1.04	0.97-1.12	0	0.30
Hyperglycaemia	1	32/88	36.36%	37/86	43.02%	39.66%	0.85	0.58-1.22	–	0.37
Leucopenia	2	101/253	39.92%	96/254	37.80%	38.86%	1.04	0.91-1.19	0	0.61
Neutropenia	5	823/2054	40.09%	484/1376	35.17%	38.12%	1.07	0.98-1.17	29	0.12
Mucositis	1	32/88	36.36%	33/86	38.37%	37.36%	0.95	0.64-1.39	–	0.78
Diarrhea	5	510/1557	32.76%	422/1276	33.07%	32.90%	1.07	0.88-1.29	67	0.52
Peripheral sensory neuropathy	4	241/773	31.18%	281/886	31.72%	31.46%	1.12	0.99-1.28	50	0.30
Nail discolouration	2	71/253	28.06%	72/254	28.35%	28.21%	0.96	0.74-1.25	0	0.79
Taste and smell disorders	1	25/88	28.41%	24/86	27.91%	28.16%	1.02	0.63-1.64	–	0.94
Vertigo	1	24/88	27.27%	22/86	25.58%	26.44%	1.07	0.65-1.75	–	0.80
Aspartate aminotransferase increased	2	78/253	30.83%	54/254	21.26%	26.04%	1.44	1.07-1.92	0	0.01
Constipation	4	378/1488	25.40%	278/1095	25.39%	25.40%	1.03	0.90-1.19	0	0.62
Headache	3	187/704	26.56%	158/705	22.41%	24.49%	1.18	0.99-1.42	0	0.07
Vomiting	5	384/1557	24.66%	258/1276	20.22%	22.66%	1.22	1.06-1.40	14	0.006
Sleep disturbance	1	22/88	25.00%	17/86	19.77%	22.41%	1.26	0.72-2.21	–	0.41
Anorexia	1	20/88	22.73%	19/86	22.09%	22.41%	1.03	0.59-1.79	–	0.92
Rash	5	456/1919	23.76%	262/1315	19.92%	22.20%	1.17	1.02-1.34	10	0.03
Cough	3	174/704	24.72%	132/705	18.72%	21.72%	1.32	1.08-1.61	0	0.007
Elevated alanine aminotransferase level	5	451/2054	21.96%	265/1376	19.26%	20.87%	1.10	0.97-1.26	0	0.15
Arthralgia	3	146/704	20.74%	148/705	20.99%	20.87%	0.95	0.70-1.28	54	0.74
Myalgia	3	150/704	21.31%	132/705	18.72%	20.01%	1.14	0.93-1.40	0	0.22
Asthenia	3	286/1400	20.43%	185/1009	18.33%	19.55%	1.02	0.86-1.20	0	0.82
Decreased neutrophil count	5	404/2035	19.85%	270/1471	18.35%	19.22%	0.98	0.76-1.27	61	0.90
Stomatitis	2	54/253	21.34%	43/254	16.93%	19.13%	1.26	0.88-1.81	0	0.20
Peripheral neuropathy	5	304/1557	19.52%	230/1276	18.03%	18.85%	1.01	0.87-1.18	28	0.87
Decreased appetite	2	118/616	19.16%	113/619	18.26%	18.70%	1.05	0.83-1.32	0	0.69
Epistaxis	2	46/253	18.18%	45/254	17.72%	17.95%	1.03	0.55-1.90	63	0.94
Hot flush	2	49/253	19.37%	41/254	16.14%	17.75%	1.19	0.82-1.72	0	0.36
Bone pain	1	17/88	19.32%	13/86	15.12%	17.24%	1.28	0.66-2.47	–	0.47
Fever without neutropenia	1	16/88	18.18%	12/86	13.95%	16.09%	1.30	0.66-2.59	–	0.45
Pyrosis	1	18/88	20.45%	10/86	11.63%	16.09%	1.76	0.86-3.59	–	0.12
Dyspnoea	3	121/704	17.19%	104/705	14.75%	15.97%	1.16	0.91-1.47	0	0.22
Hand–foot-syndrome	1	11/88	12.50%	16/86	18.60%	15.52%	0.67	0.33-1.36	–	0.27
Pyrexia	2	122/616	19.81%	69/619	11.15%	15.47%	1.78	1.35-2.34	0	<0.0001
Peripheral edema	3	111/704	15.77%	105/705	14.89%	15.33%	1.06	0.83-1.35	0	0.66
Dermatitis	1	13/88	14.77%	12/86	13.95%	14.37%	1.06	0.51-2.19	–	0.88
Insomnia	2	90/616	14.61%	81/619	13.09%	13.85%	1.12	0.85-1.48	29	0.45
Pruritus	3	111/685	16.20%	91/800	11.38%	13.60%	1.57	1.00-2.49	66	0.05
Dysgeusia	2	81/616	13.15%	84/619	13.57%	13.36%	0.90	0.54-1.48	57	0.68
Back pain	3	92/704	13.07%	90/705	12.77%	12.92%	1.02	0.78-1.34	50	0.87
Infusion reaction	3	152/1037	14.66%	55/644	8.54%	12.31%	1.55	1.16-2.08	0	0.003
Dizziness	1	64/451	14.19%	46/451	10.20%	12.20%	1.39	0.97-1.99	–	0.07
Urinary tract infection	1	56/451	12.42%	46/451	10.20%	11.31%	1.22	0.84-1.76	–	0.29
Lacrimation increased	2	30/253	11.86%	27/254	10.63%	11.24%	1.11	0.68-1.82	–	0.66
Dyspepsia	1	16/165	9.70%	21/168	12.50%	11.11%	0.78	0.42-1.43	–	0.42
Paronychia	1	15/165	9.09%	21/168	12.50%	10.81%	0.73	0.39-1.36	–	0.32
Pain in extremity	2	71/616	11.53%	62/619	10.02%	10.77%	1.15	0.83-1.59	0	0.39
Abdominal pain	3	67/704	9.52%	77/705	10.92%	10.22%	0.87	0.64-1.19	0	0.39
Upper respiratory tract infection	1	18/165	10.91%	16/168	9.52%	10.21%	1.15	0.61-2.17	–	0.68

ICIs, immune checkpoint inhibitors; CI, confidence interval.

For Grade 3–5 AEs, more cases of diarrhea, severe skin reactions, pneumonitis, hepatitis, and adrenal insufficiencies were related to the ICI+chemotherapy group. Grade 3–5 AEs greater than 1% are summarized in **Table 5**.

**Table 5:** Grade 3–5 adverse events an incidence of more than 1% according to combination of two groups.

Adverse events	Studies involved	ICIs+Chemotherapy		Chemotherapy		Total incidence	Risk ratio	95% CI	*I*2 (%)	*P*
		Event/total	%	Event/total	%					
Neutropenia	5	547/2,054	26.64%	319/1,376	23.18%	25.26%	1.02	0.91–1.15	0	0.69
Leukopenia	2	44/253	17.39%	38/254	14.96%	16.17%	1.14	0.79–1.66	39	0.48
Decreased neutrophil count	5	287/2,035	14.10%	181/1,471	12.51%	13.43%	0.90	0.76–1.06	16	0.20
Anemia	6	269/2,123	12.62%	136/1,557	8.73%	10.98%	1.17	0.96–1.42	0	0.11
Febrile neutropenia	3	28/322	8.70%	30/435	6.90%	7.66%	1.28	0.77–2.12	0	0.34
Infection	1	5/88	5.68%	4/86	4.65%	5.17%	1.22	0.34–4.40	–	0.76
Elevated alanine aminotransferase level	5	96/2,054	4.67%	44/1,376	3.20%	4.08%	1.39	0.97–1.99	0	0.08
Bone pain	1	4/88	4.55%	2/86	2.33%	3.45%	1.95	0.37–10.39	–	0.43
Fatigue	6	76/2,123	3.58%	43/1,557	2.76%	3.23%	1.36	0.94–1.97	44	0.11
Hypertension	2	14/616	2.27%	23/619	3.72%	3.00%	0.62	0.32–1.18	30	0.14
Peripheral sensory neuropathy	4	24/773	3.10%	24/886	2.71%	2.89%	1.07	0.62–1.87	0	0.80
Aspartate aminotransferase increased	2	10/253	3.95%	3/254	1.18%	2.56%	3.03	0.91–10.04	0	0.07
Peripheral neuropathy	5	46/1,557	2.95%	25/1,276	1.96%	2.51%	1.58	0.98–2.56	26	0.06
Nail discoloration	2	8/253	3.16%	4/254	1.57%	2.37%	1.86	0.61–5.71	26	0.28
Hand–foot-syndrome	1	1/88	1.14%	3/86	3.49%	2.30%	0.33	0.03–3.07	–	0.33
Nausea	6	46/2,123	2.17%	31/1,557	1.99%	2.09%	0.96	0.33–2.73	67	0.93
Diarrhea	5	37/1,557	2.38%	19/1,276	1.49%	1.98%	1.76	1.01–3.04	7	0.04
Asthenia	3	31/1,400	2.21%	15/1,009	1.49%	1.91%	1.26	0.67–2.35	0	0.47
Hypokalemia	1	11/451	2.44%	4/451	0.89%	1.66%	2.75	0.88–8.57	–	0.08
Infusion reaction	3	21/1,037	2.03%	5/644	0.78%	1.55%	2.26	0.84–6.06	0	0.11
Vomiting	5	26/1,557	1.67%	13/1,276	1.02%	1.38%	1.38	0.72–2.67	0	0.34
Severe skin reaction	5	45/2,320	1.93%	2/1,428	0.14%	1.25%	8.50	2.54–28.46	0	0.0005
Fever without neutropenia	1	1/88	1.14%	1/86	1.16%	1.15%	0.98	0.06–15.38	–	0.99
Injury-poisoning and procedure	1	1/88	1.14%	1/86	1.16%	1.15%	0.98	0.06–15.38	–	0.99
Anorexia	1	1/88	1.14%	1/86	1.16%	1.15%	0.98	0.06–15.38	–	0.99
Mucositis	1	2/88	2.27%	0/86	0.00%	1.15%	4.89	0.24–100.35	–	0.30

ICIs, immune checkpoint inhibitors; CI, confidence interval.

### Sensitivity Analysis

In the analysis of complete response, PFSR, and AEs, the *I^2^* statistic was >50%, which suggests significant heterogeneity. By removing each study, the sensitivity analysis suggested that the results were stable and reliable (**Figure S4**).

### Publication Bias

No significant publication bias was found based on the funnel plots of survival (**Figure S5A**) and safety (**Figure S5B**).

## Discussion

Due to the lack of targets for therapeutic intervention, the treatment of TNBC is challenging (23). Whether immunotherapy can improve the curative effect when added to original standard chemotherapy treatment is still controversial (7, 8). This meta-analysis first compared ICI+chemotherapy with chemotherapy for TNBC treatment. The results suggest that ICI+chemotherapy treatment showed better efficacy in OS, PFS, and complete response. With the prolongation of survival, the survival advantage of ICI+chemotherapy increased compared with that of chemotherapy. In the toxicity analysis, more Grade 3–5 AEs and serious AEs were found in the ICI+chemotherapy group.

Better survival rates were the main benefit for the ICIs+Chemotherapy group. With the prolongation of survival, the advantage of OS and PFS in the ICIs+Chemotherapy group increased compared with the chemotherapy group. Similar results were confirmed by three large sample RCTs (KEYNOTE-522, IMpassion130 and KEYNOTE-355) (9, 10, 12). The I-SPY2 study and KEYNOTE-522 study suggested that significantly higher rates of CR were achieved in the ICIs+Chemotherapy groups (9, 13). Two reasons may explain the benefit of ICIs+Chemotherapy: (1) ICIs kill tumor cells by activating tumor immunity, which is different from chemotherapy and plays a synergistic role, especially in PD-L1-positive TNBC (9, 24). The antitumor effect may be more significant in early breast cancer than metastatic disease, because the tumor immune microenvironment is more robust (25); and (2) higher CR rates (584/1,106 *vs.* 341/825, RR: 1.38, [1.01–1.89]) were found in the ICIs+Chemotherapy groups, which is very important for the long-term survival of breast cancer patients after surgery (11, 13). Cortazar et al.’s pooled analysis also confirmed the strong association of PCR (no tumor in either breast or the lymph nodes) after neoadjuvant chemotherapy with an improved long-term benefit with respect to OS and DFS, especially in patients with TNBC (26). However, subgroup analysis suggested that addition of ICIs might not have a better effect in Asian patients, patients with locally advanced disease, or patients with brain metastases. Therefore, we suggested that ICIs+Chemotherapy is better than chemotherapy alone with longer survival, especially for patients with PD-L1-positive TNBC.

Higher rate of AEs, especially Grade 3–5/serious AEs, is the main restrictive factor to add immunotherapy to chemotherapy (9, 10). Twenty-one Grade 3–5 AEs greater >2% were reported in the ICIs+Chemotherapy group (neutropenia, leukopenia, decreased neutrophil count, anemia, febrile neutropenia, infection, elevated alanine aminotransferase [ALT] levels, bone pain, increased AST levels, fatigue, nail discoloration, peripheral sensory neuropathy, peripheral neuropathy, hypokalemia, diarrhea, mucositis, hypertension, severe skin reactions, asthenia, nausea, and infusion reactions) compared with twelve in the chemotherapy group (neutropenia, leukopenia, decreased neutrophil count, anemia, febrile neutropenia, infection, hypertension, hand–foot-syndrome, elevated ALT levels, fatigue, peripheral sensory neuropathy, and bone pain). The frequency of AEs was similar as previously reported by Schmid et al. in the updated report of the IMpassion130 trial (23). Hypothyroidism, hyperthyroidism, pneumonitis, hepatitis, and adrenal insufficiency were five AEs of special interest, which were all significantly increased after the addition of ICIs (27). High levels of AEs leading to treatment discontinuation was found in the ICIs+Chemotherapy group (16.38 *vs.* 10.27%), which might decrease antitumor efficacy (10). In the subgroup analysis according to the organs, the addition of ICIs might have a greater impact on the gastrointestinal system, hepatobiliary system, respiratory system, and the thyroid. Therefore, we suggested that although ICIs+Chemotherapy has better survival efficacy, the increase in serious complications deserves attention to improve the lifelong treatment of patients during survival.

However, this meta-analysis had some limitations described as follows: (1) The treatments used in the ICIs+Chemotherapy group and chemotherapy group were different between the groups, which might also increase heterogeneity. (2) Four out of the eight included studies (9, 11, 13, 14) focused on neoadjuvant therapy for early breast cancers, and the other 4/8 studies (10, 12, 16, 18) focused on medical therapy for metastatic breast cancers, and the combined analysis might increase heterogeneity. (3) Only RCTs published in English were included, which might introduce language bias; and (4) significant heterogeneity was found in some analyses (CR, PFSR, etc.), which might decrease the credibility of these results.

ICIs+Chemotherapy appears to be better than chemotherapy alone for TNBC with better OS and PFS. With the prolonged survival time, ICIs+Chemotherapy had an increased advantage for survival. However, the high rates of Grade 3–5/serious AEs, especially immunotherapy-related AEs, need to be taken seriously. However, due to the limitations described above, the results must be confirmed by more large-sample and high-quality RCTs.

## Data Availability Statement

The original contributions presented in the study are included in the article/**Supplementary Material**, further inquiries can be directed to the corresponding author.

## Author Contributions

JH had full access to all of the data in the manuscript and takes responsibility for the integrity of the data and the accuracy of the data analysis. All authors contributed to the article and approved the submitted version. Concept and design: All authors. Acquisition, analysis, or interpretation of data: All authors. Drafting of the manuscript: QJ, JD, MH, and NL. Critical revision of the manuscript for important intellectual content: QJ, JH, and WZ. Statistical analysis: QJ, JH, and WZ. Supervision: QJ and JH.

## Funding

This study was supported by Science and Technology Planning Project of Health Commission of Jiangxi Province (Grant number: 20204261) and National Natural Science Foundation of China (NSFC, number of grants: 81560345). Role of the Funding: The funding had no role in the design and conduct of the study; collection, management, analysis, and interpretation of the data; preparation, review, or approval of the manuscript; and decision to submit the manuscript for publication.

## Conflict of Interest

The authors declare that the research was conducted in the absence of any commercial or financial relationships that could be construed as a potential conflict of interest.

## Publisher’s Note

All claims expressed in this article are solely those of the authors and do not necessarily represent those of their affiliated organizations, or those of the publisher, the editors and the reviewers. Any product that may be evaluated in this article, or claim that may be made by its manufacturer, is not guaranteed or endorsed by the publisher.

## Supplementary Material

Supplementary Table 1 PRISMA 2009 checklist.

Supplementary Table 2 Search strategies.

Supplementary Table 3 Quality assessment of the included studies according to Jadad scale.

Supplementary Table 4 GRADE quality assessment for the outcomes of survival, responses, summary of adverse events, total adverse events, and grade 3–5 adverse events.

Supplementary Figure 1 Cochrane risk assessment associated with ICIs+Chemotherapy *versus* Chemotherapy.

Supplementary Figure 2 Forest plots of OSR (6–36 months) associated with ICIs+Chemotherapy *versus* Chemotherapy according to survival time.

Supplementary Figure 3 Forest plots of PFSR (6–30 months) associated with ICIs+Chemotherapy *versus* Chemotherapy according to survival time.

Supplementary Figure 4 Sensitivity analysis of complete response **(A)**, PFSR **(B)**, and total adverse events **(C)** associated with ICIs+Chemotherapy *versus* Chemotherapy.

Supplementary Figure 5 Funnel plots of survival summary **(A)** and safety summary **(B)** associated with crizotinib *versus* alectinib.

## Abbreviations

AEs, adverse effects; ALT, alanine aminotransferase; AST, aspartate aminotransferase; CR, complete response; CRBT, Cochrane Risk of Bias Tool; ECOG, Eastern Cooperative Oncology Group; GRADE, Grading of Recommendations Assessment, Development and Evaluation; HR, Hazard ratio; HRD, homologous recombination deficiency; ICIs, immune checkpoint inhibitors; OS, overall survival; OSR, overall survival rate; PCR, pathological complete response; PFS, progression-free survival; PFSR, progression-free survival rate; PRISMA, Preferred Reporting Items for Systematic Review and Meta-Analysis guidelines; RCTs, randomized controlled trials; RR, risk ratio; TNBC, triple-negative breast cancer.
