# Supplementary material for: Immune Checkpoint Inhibitors Combined With Chemotherapy Compared With Chemotherapy Alone for Triple-Negative Breast Cancer: A Systematic Review and Meta-Analysis
Source: Front Oncol. 2021 Dec 16;11:795650. doi: 10.3389/fonc.2021.795650 (PMC8716854; doi:10.3389/fonc.2021.795650)
Supplement: Supplementary Table 2 — Search strategies. [file Table_2.docx]

**Table S2** Search strategy

| **PubMed**  The database was searched on November 13, 2021, n=215.  Search Strategy:  (Breast cancer[Title/Abstract] OR Breast Neoplasm[Title/Abstract] OR Neoplasm, Breast[Title/Abstract] OR Breast Tumor[Title/Abstract] OR Tumor, Breast[Title/Abstract] OR Cancer, Breast[Title/Abstract] OR Breast Carcinoma[Title/Abstract] OR Carcinoma, Breast[Title/Abstract]) AND (Alectinib[Title/Abstract] OR RO5424802[Title/Abstract] OR CH5424802[Title/Abstract] OR Alecensa[Title/Abstract]) AND (Immune checkpoint inhibitor[Title/Abstract] OR Checkpoint Inhibitor, Immune[Title/Abstract] OR Immune Checkpoint Blocker[Title/Abstract] OR Immune Checkpoint Blockade[Title/Abstract] OR PD-L1 Inhibitor[Title/Abstract] OR CTLA-4 Inhibitor[Title/Abstract] OR PD-1 Inhibitor[Title/Abstract]) AND (Chemotherapy[Title/Abstract] OR Drug Therapy[Title/Abstract] OR Chemotherapies[Title/Abstract] OR Pharmacotherapy[Title/Abstract]) |
| --- |
| **Web of Science**  The database was searched on November 13, 2021, n=217.  Search Strategy:  1 TOPIC: (“Breast cancer” OR “Breast Neoplasm” OR “Neoplasm, Breast” OR “Breast Tumor” OR “Tumor, Breast” OR “Cancer, Breast” OR “Breast Carcinoma” OR “Carcinoma, Breast”) (542598)  2 TOPIC: (“Immune checkpoint inhibitor” OR “Checkpoint Inhibitor, Immune” OR “Immune Checkpoint Blocker” or “Immune Checkpoint Blockade” OR “PD-L1 Inhibitor” OR “CTLA-4 Inhibitor” OR “PD-1 Inhibitor”) (10087)  3 TOPIC: (“Chemotherapy” OR “Drug Therapy” OR “Chemotherapies” OR “Pharmacotherapy”) (555009)  4 #1 AND #2 AND #3 (204) |
| **EMBASE**  The database was searched on November 13, 2021, n=523.  Search Strategy:  ('Breast cancer':ti,ab,kw OR 'Breast Neoplasm':ti,ab,kw OR 'Neoplasm, Breast':ti,ab,kw OR 'Breast Tumor':ti,ab,kw OR 'Tumor, Breast':ti,ab,kw OR 'Cancer, Breast':ti,ab,kw OR 'Breast Carcinoma':ti,ab,kw OR 'Carcinoma, Breast':ti,ab,kw) AND ('Immune checkpoint inhibitor':ti,ab,kw OR 'Checkpoint Inhibitor, Immune':ti,ab,kw OR 'Immune Checkpoint Blocker':ti,ab,kw OR 'Immune Checkpoint Blockade':ti,ab,kw OR 'PD-L1 Inhibitor':ti,ab,kw OR 'CTLA-4 Inhibitor':ti,ab,kw OR 'PD-1 Inhibitor':ti,ab,kw) AND ('Chemotherapy':ti,ab,kw OR 'Drug Therapy':ti,ab,kw OR 'Chemotherapies':ti,ab,kw OR 'Pharmacotherapy':ti,ab,kw) |
| **Cochrane Library**  The database was searched on November 13, 2021, n=62.  Search Strategy:  (“ Breast cancer ” OR “ Breast Neoplasm” OR “Neoplasm, Breast” OR “Breast Tumor” OR “Tumor, Breast” OR “Cancer, Breast” OR “Breast Carcinoma” OR “Carcinoma, Breast”): ti,ab,kw AND (“Immune checkpoint inhibitor” OR “Checkpoint Inhibitor, Immune” OR “Immune Checkpoint Blockade” or “PD-L1 Inhibitor” OR “CTLA-4 Inhibitor” OR “PD-1 Inhibitor”): ti,ab,kw AND (“Chemotherapy” OR “Drug Therapy” OR “Chemotherapies” OR “Pharmacotherapy”): ti,ab,kw - (Word variations have been searched) |
| **Ovid MEDLINE**  The database was searched on November 13, 2021, n=867.  Search Strategy:  1 Breast cancer .ab. (10115)  2 Breast Neoplasm.ab. (10840)  3 Neoplasm, Breast.ab. (16033)  4 Breast Tumor.ab. (8736)  5 Tumor, Breast.ab. (15963)  6 Cancer, Breast.ab. (14668)  7 Breast Carcinoma.ab. (11102)  8 Carcinoma, Breast.ab. (16336)  9 or/1-9 [ Breast cancer ] (52369)  10 Immune checkpoint inhibitor.ab. (5688)  11 Checkpoint Inhibitor, Immune.ab. (9685)  12 Immune Checkpoint Blocker.ab. (7125)  13 Immune Checkpoint Blockade.ab. (5968)  14 PD-L1 Inhibitor.ab. (5863)  15 CTLA-4 Inhibitor.ab. (2968)  16 PD-1 Inhibitor.ab. (6555)  17 or/10-16 [Immune checkpoint inhibitor] (22568)  18 Chemotherapy.ab. ( 3298 )  19 Drug Therapy.ab. (5587)  20 Chemotherapies.ab. (22136)  21 Pharmacotherapy.ab. (5539)  22 or/18-21 [Lung cancer] (29356)  23 9 and 17 and22 (867) |
| **ScienceDirect**  The database was searched on November 13, 2021, n=82.  Search Strategy:  Title, abstract, keywords: ((“Breast cancer ” OR “ Breast Neoplasm” OR “Neoplasm, Breast” OR “Breast Tumor” OR “Tumor, Breast” OR “Cancer, Breast” OR “Breast Carcinoma” OR “Carcinoma, Breast”) and (“Immune checkpoint inhibitor” OR “Checkpoint Inhibitor, Immune” OR “Immune Checkpoint Blockade” or “PD-L1 Inhibitor” OR “CTLA-4 Inhibitor” OR “PD-1 Inhibitor”) and (“Chemotherapy” OR “Drug Therapy” OR “Chemotherapies” OR “Pharmacotherapy”)) |
| **Scopus**  The database was searched on November 13, 2021, n=736.  Search Strategy:  TITLE-ABS-KEY ((“Breast cancer ” OR “ Breast Neoplasm” OR “Neoplasm, Breast” OR “Breast Tumor” OR “Tumor, Breast” OR “Cancer, Breast” OR “Breast Carcinoma” OR “Carcinoma, Breast”) and (“Immune checkpoint inhibitor” OR “Checkpoint Inhibitor, Immune” OR “Immune Checkpoint Blockade” or “PD-L1 Inhibitor” OR “CTLA-4 Inhibitor” OR “PD-1 Inhibitor”) and (“Chemotherapy” OR “Drug Therapy” OR “Chemotherapies” OR “Pharmacotherapy”)) |

**Note:** The combined text and medical subject heading (MeSH) terms used were: “Breast cancer”, “Immune checkpoint inhibitor” and “Chemotherapy”.
