# Supplementary material for: Immune Checkpoint Inhibitors Combined With Chemotherapy Compared With Chemotherapy Alone for Triple-Negative Breast Cancer: A Systematic Review and Meta-Analysis
Source: Front Oncol. 2021 Dec 16;11:795650. doi: 10.3389/fonc.2021.795650 (PMC8716854; doi:10.3389/fonc.2021.795650)
Supplement: Supplementary Table 3 — Quality assessment of the included studies according to Jadad scale. [file Table_3.doc]

**Table S3 Quality assessment of the included studies according to the Jadad scale.**

| **Study** | | | **Randomization** | **Masking** | **Accountability of all patients** | **Quality (score)** |
| --- | --- | --- | --- | --- | --- | --- |
| 2021 | Miles [15] | IMpassion131 | ** | ** | * | 5 |
| 2021 | Bachelot [16] | SAFIR02-BREAST IMMUNO | ** | ** | * | 5 |
| 2020 | Schmid [9] | KEYNOTE-522 | ** | ** | * | 5 |
| 2020 | Schmid [10] | IMpassion130 | ** | ** | * | 5 |
| 2020 | Mittendorf [11] | IMpassion031 | ** | ** | * | 5 |
| 2020 | Cortes [12] | KEYNOTE-355 | ** | ** | * | 5 |
| 2020 | Nanda [13] | I-SPY2 | ** | ** | * | 5 |
| 2020 | Tolaney[18] | - | * | ** | * | 4 |
| 2019 | Loibl [14] | GeparNuevo | ** | ** | * | 5 |
