# Supplementary material for: Immune Checkpoint Inhibitors Combined With Chemotherapy Compared With Chemotherapy Alone for Triple-Negative Breast Cancer: A Systematic Review and Meta-Analysis
Source: Front Oncol. 2021 Dec 16;11:795650. doi: 10.3389/fonc.2021.795650 (PMC8716854; doi:10.3389/fonc.2021.795650)
Supplement: Supplementary Table 4 — GRADE quality assessment for the outcomes of survival, responses, summary of adverse events, total adverse events, and grade 3–5 adverse events. [file Table_4.doc]

**Table S4** GRADE quality assessment for the outcomes of survival, responses, summary of adverse events, total adverse events and grade 3-5 adverse events.

| **Primary outcomes** | **No. of Studies** | **No. of Participants** | | **Differences（95%CI）** | **Quality Assessment** | | | | | **Quality** |
| --- | --- | --- | --- | --- | --- | --- | --- | --- | --- | --- |
|  |  | **ICIs+Chemotherapy** | **Chemotherapy** |  | **Risk of Bias** | **Inconsistency** | **Indirectness** | **Imprecision** | **Publication Bias** |  |
| **Survival** |  |  |  |  |  |  |  |  |  |  |
| OS | 5 | 1138 | 918 | 0.86 [0.74, 0.99] | Low | No inconsistency | No indirectness | No imprecision | Unlikely | High |
| OSR |  |  |  |  |  |  |  |  |  |  |
| 6-month | 3 | 626/626 | 482/563 | 1.17 [1.13, 1.21] | Low | No inconsistency | No indirectness | No imprecision | Unlikely | High |
| 12-month | 3 | 448/626 | 372/563 | 1.08 [1.00, 1.17] | Low | No inconsistency | No indirectness | No imprecision | Unlikely | High |
| 18-month | 3 | 348/626 | 281/563 | 1.11 [0.99, 1.24] | Low | No inconsistency | No indirectness | No imprecision | Unlikely | High |
| 24-month | 2 | 248/582 | 198/519 | 1.12 [0.97, 1.30] | Low | No inconsistency | No indirectness | No imprecision | Unlikely | High |
| 30-month | 2 | 194/582 | 146/519 | 1.20 [1.00, 1.44] | Low | No inconsistency | No indirectness | No imprecision | Unlikely | High |
| 36-month | 2 | 147/582 | 96/519 | 1.33 [1.06, 1.67] | Low | No inconsistency | No indirectness | No imprecision | Unlikely | High |
| PFS | 6 | 2441 | 1554 | 0.78 [0.70, 0.86] | Serious (-1) | No inconsistency | No indirectness | No imprecision | Unlikely | Medium |
| PFSR |  |  |  |  |  |  |  |  |  |  |
| 6-month | 4 | 1334/1845 | 728/1166 | 1.09 [0.78, 1.52] | Low | No inconsistency | No indirectness | No imprecision | Unlikely | High |
| 12-month | 4 | 1017/1845 | 507/1166 | 1.26 [0.84, 1.88] | Low | No inconsistency | No indirectness | No imprecision | Unlikely | High |
| 18-month | 3 | 905/1801 | 428/1122 | 1.26 [0.90, 1.75] | Low | No inconsistency | No indirectness | No imprecision | Unlikely | High |
| 24-month | 3 | 849/1801 | 375/1122 | 1.35 [0.95, 1.91] | Low | No inconsistency | No indirectness | No imprecision | Unlikely | High |
| 30-month | 2 | 132/1017 | 42/732 | 2.05 [1.46, 2.86] | Serious (-1) | No inconsistency | No indirectness | No imprecision | Unlikely | Medium |
| Complete response | 4 | 584/1103 | 340/825 | 1.38 [1.01, 1.89] | Serious (-1) | No inconsistency | No indirectness | No imprecision | Unlikely | Medium |
| **Summary of adverse events** |  |  |  |  |  |  |  |  |  |  |
| Total AEs | 7 | 2462/2488 | 1550/1589 | 1.01 [0.99-1.03] | Serious (-1) | No inconsistency | No indirectness | No imprecision | Unlikely | Medium |
| Treatment-related AEs | 5 | 1951/2013 | 1255/1325 | 1.02 [0.98-1.06] | Serious (-1) | No inconsistency | No indirectness | No imprecision | Unlikely | Medium |
| Grade 3-5 AEs | 6 | 1697/2444 | 901/1545 | 1.14 [1.03-1.25] | Low | No inconsistency | No indirectness | No imprecision | Unlikely | High |
| Treatment-related grade 3-5 AEs | 6 | 1295/2057 | 724/1369 | 1.09 [1.03-1.16] | Low | No inconsistency | No indirectness | No imprecision | Unlikely | High |
| Serious AEs | 2 | 155/616 | 111/619 | 1.40 [1.13-1.74] | Low | No inconsistency | No indirectness | No imprecision | Unlikely | High |
| Treatment-related serious AEs | 4 | 128/751 | 88/740 | 1.44 [1.13-1.85] | Low | No inconsistency | No indirectness | No imprecision | Unlikely | High |
| AEs leading to treatment discontinuation | 4 | 123/751 | 76/740 | 1.61 [1.24-2.10] | Low | No inconsistency | No indirectness | No imprecision | Unlikely | High |
| AEs leading to dose reduction/dose interruption | 1 | 194/451 | 173/451 | 1.12 [0.96-1.31] | Low | No inconsistency | No indirectness | No imprecision | Unlikely | High |
| Death | 3 | 7/663 | 4/654 | 1.76 [0.52-5.97] | Low | No inconsistency | No indirectness | No imprecision | Unlikely | High |
| Treatment-related death | 1 | 2/451 | 1/451 | 2.00 [0.18-21.9] | Low | No inconsistency | No indirectness | No imprecision | Unlikely | High |
| **Total adverse events** |  |  |  |  |  |  |  |  |  |  |
| Fatigue | 6 | 882/2123 | 709/1557 | 1.04 [0.97, 1.12] | Low | No inconsistency | No indirectness | No imprecision | Unlikely | High |
| Asthenia | 3 | 286/1400 | 185/1009 | 1.02 [0.86, 1.20] | Low | No inconsistency | No indirectness | No imprecision | Unlikely | High |
| Infusion reaction | 3 | 152/1037 | 55/644 | 1.55 [1.16, 2.08] | Low | No inconsistency | No indirectness | No imprecision | Unlikely | High |
| Pyrexia | 2 | 122/616 | 69/619 | 1.78 [1.35, 2.34] | Low | No inconsistency | No indirectness | No imprecision | Unlikely | High |
| Malaise | 1 | 14/165 | 18/168 | 0.79 [0.41, 1.54] | Low | No inconsistency | No indirectness | No imprecision | Unlikely | High |
| Fever without neutropenia | 1 | 16/88 | 12/86 | 1.30 [0.66, 2.59] | Low | No inconsistency | No indirectness | No imprecision | Unlikely | High |
| Infection | 1 | 50/88 | 39/86 | 1.25 [0.93, 1.68] | Low | No inconsistency | No indirectness | No imprecision | Unlikely | High |
| Xerostomia | 1 | 7/88 | 3/86 | 2.28 [0.61, 8.53] | Low | No inconsistency | No indirectness | No imprecision | Unlikely | High |
| Injury, poisoning and procedure | 1 | 7/88 | 3/86 | 2.28 [0.61, 8.53] | Low | No inconsistency | No indirectness | No imprecision | Unlikely | High |
| Peripheral neuropathy | 5 | 304/1557 | 230/1276 | 1.01 [0.87, 1.18] | Low | No inconsistency | No indirectness | No imprecision | Unlikely | High |
| Headache | 3 | 187/704 | 158/705 | 1.18 [0.99, 1.42] | Low | No inconsistency | No indirectness | No imprecision | Unlikely | High |
| Dysgeusia | 2 | 81/616 | 84/619 | 0.90 [0.54, 1.48] | Low | No inconsistency | No indirectness | No imprecision | Unlikely | High |
| Dizziness | 1 | 64/451 | 46/451 | 1.39 [0.97, 1.99] | Low | No inconsistency | No indirectness | No imprecision | Unlikely | High |
| Peripheral sensory neuropathy | 4 | 241/773 | 281/886 | 1.12 [0.99, 1.28] | Low | No inconsistency | No indirectness | No imprecision | Unlikely | High |
| Insomnia | 2 | 90/616 | 81/619 | 1.12 [0.85, 1.48] | Low | No inconsistency | No indirectness | No imprecision | Unlikely | High |
| Paraesthesia | 1 | 7/165 | 19/168 | 0.38 [0.16, 0.87] | Low | No inconsistency | No indirectness | No imprecision | Unlikely | High |
| Guillain-Barré syndrome | 2 | 1/596 | 2/388 | 0.48 [0.07, 3.44] | Low | No inconsistency | No indirectness | No imprecision | Unlikely | High |
| Encephalitis | 1 | 1/165 | 0/168 | 3.05 [0.13, 74.4] | Low | No inconsistency | No indirectness | No imprecision | Unlikely | High |
| Vertigo | 1 | 24/88 | 22/86 | 1.07 [0.65, 1.75] | Low | No inconsistency | No indirectness | No imprecision | Unlikely | High |
| Depression | 1 | 7/88 | 8/86 | 0.86 [0.32, 2.26] | Low | No inconsistency | No indirectness | No imprecision | Unlikely | High |
| Sleep disturbance | 1 | 22/88 | 17/86 | 1.26 [0.72, 2.21] | Low | No inconsistency | No indirectness | No imprecision | Unlikely | High |
| Taste and smell disorders | 1 | 25/88 | 24/86 | 1.02 [0.63, 1.64] | Low | No inconsistency | No indirectness | No imprecision | Unlikely | High |
| Hypophysitis | 2 | 2/519 | 2/306 | 0.71 [0.10, 4.90] | Low | No inconsistency | No indirectness | No imprecision | Unlikely | High |
| Lacrimation increased | 2 | 30/253 | 27/254 | 1.11 [0.68, 1.82] | Low | No inconsistency | No indirectness | No imprecision | Unlikely | High |
| Oropharyngeal pain | 1 | 15/165 | 18/168 | 0.85 [0.44, 1.63] | Low | No inconsistency | No indirectness | No imprecision | Unlikely | High |
| Ocular inflammatory toxicity | 2 | 6/596 | 1/388 | 2.87 [0.54, 16.25] | Low | No inconsistency | No indirectness | No imprecision | Unlikely | High |
| Dry eye | 1 | 4/88 | 5/86 | 0.78 [0.22, 2.81] | Low | No inconsistency | No indirectness | No imprecision | Unlikely | High |
| Nausea | 6 | 1135/2123 | 825/1557 | 1.04 [0.98, 1.10] | Low | No inconsistency | No indirectness | No imprecision | Unlikely | High |
| Diarrhea | 5 | 510/1557 | 422/1276 | 1.07 [0.88, 1.29] | Low | No inconsistency | No indirectness | No imprecision | Unlikely | High |
| Vomiting | 5 | 384/1557 | 258/1276 | 1.22 [1.06, 1.40] | Low | No inconsistency | No indirectness | No imprecision | Unlikely | High |
| Constipation | 4 | 378/1488 | 278/1095 | 1.03 [0.90, 1.19] | Low | No inconsistency | No indirectness | No imprecision | Unlikely | High |
| Decreased appetite | 2 | 118/616 | 113/619 | 1.05 [0.83, 1.32] | Low | No inconsistency | No indirectness | No imprecision | Unlikely | High |
| Abdominal pain | 3 | 67/704 | 77/705 | 0.87 [0.64, 1.19] | Low | No inconsistency | No indirectness | No imprecision | Unlikely | High |
| Colitis | 5 | 21/1682 | 12/1301 | 1.26 [0.62, 2.56] | Low | No inconsistency | No indirectness | No imprecision | Unlikely | High |
| Dyspepsia | 1 | 16/165 | 21/168 | 0.78 [0.42, 1.43] | Low | No inconsistency | No indirectness | No imprecision | Unlikely | High |
| Anorexia | 1 | 20/88 | 19/86 | 1.03 [0.59, 1.79] | Low | No inconsistency | No indirectness | No imprecision | Unlikely | High |
| Pyrosis | 1 | 18/88 | 10/86 | 1.76 [0.86, 3.59] | Low | No inconsistency | No indirectness | No imprecision | Unlikely | High |
| Alopecia | 5 | 1123/2054 | 771/1376 | 1.03 [0.97, 1.09] | Low | No inconsistency | No indirectness | No imprecision | Unlikely | High |
| Rash | 5 | 456/1919 | 262/11315 | 1.17 [1.02, 1.34] | Low | No inconsistency | No indirectness | No imprecision | Unlikely | High |
| Severe skin reaction | 5 | 104/2320 | 52/1428 | 1.48 [1.11, 1.99] | Low | No inconsistency | No indirectness | No imprecision | Unlikely | High |
| Peripheral edema | 3 | 111/704 | 105/705 | 1.06 [0.83, 1.35] | Low | No inconsistency | No indirectness | No imprecision | Unlikely | High |
| Pruritus | 3 | 111/685 | 91/800 | 1.57 [1.00, 2.49] | Low | No inconsistency | No indirectness | No imprecision | Unlikely | High |
| Pain in extremity | 2 | 71/616 | 62/619 | 1.15 [0.83, 1.59] | Low | No inconsistency | No indirectness | No imprecision | Unlikely | High |
| Nasopharyngitis | 1 | 48/451 | 37/451 | 1.30 [0.86, 1.95] | Low | No inconsistency | No indirectness | No imprecision | Unlikely | High |
| Stomatitis | 2 | 54/253 | 43/254 | 1.26 [0.88, 1.81] | Low | No inconsistency | No indirectness | No imprecision | Unlikely | High |
| Nail discolouration | 2 | 71/253 | 72/254 | 0.96 [0.74, 1.25] | Low | No inconsistency | No indirectness | No imprecision | Unlikely | High |
| Epistaxis | 2 | 46/253 | 45/254 | 1.03 [0.55, 1.90] | Low | No inconsistency | No indirectness | No imprecision | Unlikely | High |
| Hot flush | 2 | 49/253 | 41/254 | 1.19 [0.82, 1.72] | Low | No inconsistency | No indirectness | No imprecision | Unlikely | High |
| Nail disorder | 1 | 21/165 | 10/168 | 2.14 [1.04, 4.40] | Low | No inconsistency | No indirectness | No imprecision | Unlikely | High |
| Paronychia | 1 | 15/165 | 21/168 | 0.73 [0.39, 1.36] | Low | No inconsistency | No indirectness | No imprecision | Unlikely | High |
| Mucosal inflammation | 1 | 17/165 | 15/168 | 1.15 [0.60, 2.23] | Low | No inconsistency | No indirectness | No imprecision | Unlikely | High |
| Dry skin | 2 | 27/253 | 19/254 | 1.43 [0.81, 2.50] | Low | No inconsistency | No indirectness | No imprecision | Unlikely | High |
| Mucositis | 1 | 32/88 | 33/86 | 0.95 [0.64, 1.39] | Low | No inconsistency | No indirectness | No imprecision | Unlikely | High |
| Hand–foot-syndrome | 1 | 11/88 | 16/86 | 0.67 [0.33, 1.36] | Low | No inconsistency | No indirectness | No imprecision | Unlikely | High |
| Dermatitis | 1 | 13/88 | 12/86 | 1.06 [0.51, 2.19] | Low | No inconsistency | No indirectness | No imprecision | Unlikely | High |
| Arthralgia | 3 | 146/704 | 148/705 | 0.95 [0.70, 1.28] | Low | No inconsistency | No indirectness | No imprecision | Unlikely | High |
| Back pain | 3 | 92/704 | 90/705 | 1.02 [0.78, 1.34] | Low | No inconsistency | No indirectness | No imprecision | Unlikely | High |
| Myalgia | 3 | 150/704 | 132/705 | 1.14 [0.93, 1.40] | Low | No inconsistency | No indirectness | No imprecision | Unlikely | High |
| Myositis | 2 | 3/596 | 0/388 | 2.78 [0.31, 25.08] | Low | No inconsistency | No indirectness | No imprecision | Unlikely | High |
| Bone pain | 1 | 17/88 | 13/86 | 1.28 [0.66, 2.47] | Low | No inconsistency | No indirectness | No imprecision | Unlikely | High |
| Cough | 3 | 174/704 | 132/705 | 1.32 [1.08, 1.61] | Low | No inconsistency | No indirectness | No imprecision | Unlikely | High |
| Dyspnoea | 3 | 121/704 | 104/705 | 1.16 [0.91, 1.47] | Low | No inconsistency | No indirectness | No imprecision | Unlikely | High |
| Pneumonitis | 6 | 59/1770 | 15/1387 | 3.23 [1.81, 5.74] | Low | No inconsistency | No indirectness | No imprecision | Unlikely | High |
| Upper respiratory tract infection | 1 | 18/165 | 16/168 | 1.15 [0.61, 2.17] | Low | No inconsistency | No indirectness | No imprecision | Unlikely | High |
| Hypokalaemia | 1 | 28/451 | 10/451 | 2.80 [1.38, 5.70] | Low | No inconsistency | No indirectness | No imprecision | Unlikely | High |
| Hypertension | 2 | 36/616 | 43/619 | 0.84 [0.55, 1.29] | Low | No inconsistency | No indirectness | No imprecision | Unlikely | High |
| Hypotension | 1 | 3/88 | 6/86 | 0.49 [0.13, 1.89] | Low | No inconsistency | No indirectness | No imprecision | Unlikely | High |
| Decreased neutrophil count | 5 | 404/2035 | 270/1471 | 0.98 [0.76, 1.27] | Low | No inconsistency | No indirectness | No imprecision | Unlikely | High |
| Anemia | 6 | 1004/2123 | 640/1557 | 1.05 [0.98, 1.13] | Low | No inconsistency | No indirectness | No imprecision | Unlikely | High |
| Neutropenia | 5 | 823/2054 | 484/1376 | 1.07 [0.98, 1.17] | Low | No inconsistency | No indirectness | No imprecision | Unlikely | High |
| Leucopenia | 2 | 101/253 | 96/254 | 1.04 [0.91, 1.19] | Low | No inconsistency | No indirectness | No imprecision | Unlikely | High |
| Febrile neutropenia | 3 | 28/322 | 30/435 | 1.28 [0.77, 2.12] | Low | No inconsistency | No indirectness | No imprecision | Unlikely | High |
| Thromboembolic event | 1 | 1/88 | 4/86 | 0.24 [0.03, 2.14] | Low | No inconsistency | No indirectness | No imprecision | Unlikely | High |
| Elevated alanine aminotransferase level | 5 | 451/2054 | 265/1376 | 1.10 [0.97, 1.26] | Low | No inconsistency | No indirectness | No imprecision | Unlikely | High |
| Hepatitis | 5 | 30/1204 | 11/1106 | 2.40 [1.26, 4.60] | Low | No inconsistency | No indirectness | No imprecision | Unlikely | High |
| Aspartate aminotransferase increased | 2 | 78/253 | 54/254 | 1.44 [1.07, 1.92] | Low | No inconsistency | No indirectness | No imprecision | Unlikely | High |
| Increased bilirubin | 1 | 5/88 | 6/86 | 0.81 [0.26, 2.57] | Low | No inconsistency | No indirectness | No imprecision | Unlikely | High |
| Hepatotoxicity | 1 | 7/88 | 6/86 | 1.14 [0.40, 3.26] | Low | No inconsistency | No indirectness | No imprecision | Unlikely | High |
| Adrenal insufficiency | 5 | 32/1900 | 1/1410 | 8.37 [2.79, 25.1] | Low | No inconsistency | No indirectness | No imprecision | Unlikely | High |
| Urinary tract infection | 1 | 56/451 | 46/451 | 1.22 [0.84, 1.76] | Low | No inconsistency | No indirectness | No imprecision | Unlikely | High |
| Increased creatinine | 1 | 10/88 | 7/86 | 1.40 [0.56, 3.50] | Low | No inconsistency | No indirectness | No imprecision | Unlikely | High |
| Hyperthyroidism | 7 | 128/2554 | 14/1777 | 5.73 [3.38, 9.72] | Low | No inconsistency | No indirectness | No imprecision | Unlikely | High |
| Hypothyroidism | 7 | 359/2554 | 58/1777 | 4.00 [3.06, 5.23] | Low | No inconsistency | No indirectness | No imprecision | Unlikely | High |
| Diabetes | 7 | 6/596 | 3/388 | 1.21 [0.30, 4.91] | Low | No inconsistency | No indirectness | No imprecision | Unlikely | High |
| Hyperglycaemia | 1 | 32/88 | 37/86 | 0.85 [0.58, 1.22] | Low | No inconsistency | No indirectness | No imprecision | Unlikely | High |
| **Grade 3-5 adverse events** |  |  |  |  |  |  |  |  |  |  |
| Fatigue | 6 | 76/2123 | 43/1557 | 1.36 [0.94, 1.97] | Low | No inconsistency | No indirectness | No imprecision | Unlikely | High |
| Asthenia | 3 | 31/1400 | 15/1009 | 1.26 [0.67, 2.35] | Low | No inconsistency | No indirectness | No imprecision | Unlikely | High |
| Infusion reaction | 3 | 21/1037 | 5/644 | 2.26 [0.84, 6.06] | Low | No inconsistency | No indirectness | No imprecision | Unlikely | High |
| Pyrexia | 2 | 5/616 | 0/619 | 6.05 [0.73, 50.1] | Low | No inconsistency | No indirectness | No imprecision | Unlikely | High |
| Malaise | 1 | 1/165 | 0/168 | 3.05 [0.13, 74.4] | Low | No inconsistency | No indirectness | No imprecision | Unlikely | High |
| Fever without neutropenia | 1 | 1/88 | 1/86 | 0.98 [0.06, 15.3] | Low | No inconsistency | No indirectness | No imprecision | Unlikely | High |
| Infection | 1 | 5/88 | 4/86 | 1.22 [0.34, 4.40] | Low | No inconsistency | No indirectness | No imprecision | Unlikely | High |
| Xerostomia | 1 | 0/88 | 0/86 | Not estimable | Low | No inconsistency | No indirectness | No imprecision | Unlikely | High |
| Injury, poisoning and procedure | 1 | 1/88 | 1/86 | 0.98 [0.06, 15.3] | Low | No inconsistency | No indirectness | No imprecision | Unlikely | High |
| Peripheral neuropathy | 5 | 46/1557 | 25/1276 | 1.58 [0.98, 2.56] | Low | No inconsistency | No indirectness | No imprecision | Unlikely | High |
| Headache | 3 | 3/704 | 5/705 | 0.67 [0.19, 2.36] | Low | No inconsistency | No indirectness | No imprecision | Unlikely | High |
| Dysgeusia | 2 | 0/616 | 0/619 | Not estimable | Low | No inconsistency | No indirectness | No imprecision | Unlikely | High |
| Dizziness | 1 | 0/451 | 0/451 | Not estimable | Low | No inconsistency | No indirectness | No imprecision | Unlikely | High |
| Peripheral sensory neuropathy | 4 | 24/773 | 24/886 | 1.07 [0.62, 1.87] | Low | No inconsistency | No indirectness | No imprecision | Unlikely | High |
| Insomnia | 2 | 0/616 | 3/619 | 0.14 [0.01, 2.76] | Low | No inconsistency | No indirectness | No imprecision | Unlikely | High |
| Paraesthesia | 1 | 0/165 | 0/168 | Not estimable | Low | No inconsistency | No indirectness | No imprecision | Unlikely | High |
| Guillain-Barré syndrome | 2 | 1/596 | 1/388 | 0.71 [0.09, 5.83] | Low | No inconsistency | No indirectness | No imprecision | Unlikely | High |
| Encephalitis | 1 | 1/165 | 0/168 | 3.05 [0.13, 74.4] | Low | No inconsistency | No indirectness | No imprecision | Unlikely | High |
| Vertigo | 1 | 0/88 | 0/86 | Not estimable | Low | No inconsistency | No indirectness | No imprecision | Unlikely | High |
| Depression | 1 | 0/88 | 1/86 | 0.33 [0.01, 7.89] | Low | No inconsistency | No indirectness | No imprecision | Unlikely | High |
| Sleep disturbance | 1 | 0/88 | 1/86 | 0.33 [0.01, 7.89] | Low | No inconsistency | No indirectness | No imprecision | Unlikely | High |
| Taste and smell disorders | 1 | 0/88 | 0/86 | Not estimable | Low | No inconsistency | No indirectness | No imprecision | Unlikely | High |
| Hypophysitis | 2 | 0/519 | 0/306 | 0.17 [0.01, 4.17] | Low | No inconsistency | No indirectness | No imprecision | Unlikely | High |
| Lacrimation increased | 2 | 0/253 | 0/254 | Not estimable | Low | No inconsistency | No indirectness | No imprecision | Unlikely | High |
| Oropharyngeal pain | 1 | 1/165 | 0/168 | 3.05 [0.13, 74.4] | Low | No inconsistency | No indirectness | No imprecision | Unlikely | High |
| Ocular inflammatory toxicity | 2 | 0/596 | 0/388 | Not estimable | Low | No inconsistency | No indirectness | No imprecision | Unlikely | High |
| Dry eye | 1 | 0/88 | 0/86 | Not estimable | Low | No inconsistency | No indirectness | No imprecision | Unlikely | High |
| Nausea | 6 | 46/2123 | 31/1557 | 0.96 [0.33, 2.73] | Low | No inconsistency | No indirectness | No imprecision | Unlikely | High |
| Diarrhea | 5 | 37/1557 | 19/1276 | 1.76 [1.01, 3.04] | Low | No inconsistency | No indirectness | No imprecision | Unlikely | High |
| Vomiting | 5 | 26/1557 | 13/1276 | 1.38 [0.72, 2.67] | Low | No inconsistency | No indirectness | No imprecision | Unlikely | High |
| Constipation | 4 | 4/1488 | 3/1095 | 1.28 [0.32, 5.18] | Low | No inconsistency | No indirectness | No imprecision | Unlikely | High |
| Decreased appetite | 2 | 3/616 | 3/619 | 1.00 [0.20, 4.93] | Low | No inconsistency | No indirectness | No imprecision | Unlikely | High |
| Abdominal pain | 3 | 2/704 | 2/705 | 1.01 [0.17, 5.81] | Low | No inconsistency | No indirectness | No imprecision | Unlikely | High |
| Colitis | 5 | 6/1682 | 4/1301 | 1.17 [0.39, 3.57] | Low | No inconsistency | No indirectness | No imprecision | Unlikely | High |
| Dyspepsia | 1 | 0/165 | 0/168 | Not estimable | Low | No inconsistency | No indirectness | No imprecision | Unlikely | High |
| Anorexia | 1 | 1/88 | 1/86 | 0.98 [0.06, 15.3] | Low | No inconsistency | No indirectness | No imprecision | Unlikely | High |
| Pyrosis | 1 | 0/88 | 0/86 | Not estimable | Low | No inconsistency | No indirectness | No imprecision | Unlikely | High |
| Alopecia | 5 | 21/2054 | 12/1376 | 0.93 [0.46, 1.87] | Low | No inconsistency | No indirectness | No imprecision | Unlikely | High |
| Rash | 5 | 18/1919 | 7/1315 | 1.80 [0.74, 4.35] | Low | No inconsistency | No indirectness | No imprecision | Unlikely | High |
| Severe skin reaction | 5 | 45/2320 | 2/1428 | 8.50 [2.54, 28.5] | Low | No inconsistency | No indirectness | No imprecision | Unlikely | High |
| Peripheral edema | 3 | 2/704 | 8/705 | 0.29 [0.07, 1.20] | Low | No inconsistency | No indirectness | No imprecision | Unlikely | High |
| Pruritus | 3 | 1/685 | 5/800 | 0.36 [0.06, 2.06] | Low | No inconsistency | No indirectness | No imprecision | Unlikely | High |
| Pain in extremity | 2 | 2/616 | 2/619 | 1.01 [0.17, 5.81] | Low | No inconsistency | No indirectness | No imprecision | Unlikely | High |
| Nasopharyngitis | 1 | 0/451 | 0/451 | Not estimable | Low | No inconsistency | No indirectness | No imprecision | Unlikely | High |
| Stomatitis | 2 | 3/253 | 0/254 | 3.98 [0.45, 35.4] | Low | No inconsistency | No indirectness | No imprecision | Unlikely | High |
| Nail discolouration | 2 | 8/253 | 4/254 | 1.86 [0.61, 5.71] | Low | No inconsistency | No indirectness | No imprecision | Unlikely | High |
| Epistaxis | 2 | 0/253 | 0/254 | Not estimable | Low | No inconsistency | No indirectness | No imprecision | Unlikely | High |
| Hot flush | 2 | 1/253 | 3/254 | 0.33 [0.03, 3.07] | Low | No inconsistency | No indirectness | No imprecision | Unlikely | High |
| Nail disorder | 1 | 0/165 | 0/168 | Not estimable | Low | No inconsistency | No indirectness | No imprecision | Unlikely | High |
| Paronychia | 1 | 0/165 | 0/168 | Not estimable | Low | No inconsistency | No indirectness | No imprecision | Unlikely | High |
| Mucosal inflammation | 1 | 1/165 | 0/168 | 3.05 [0.13, 74.4] | Low | No inconsistency | No indirectness | No imprecision | Unlikely | High |
| Dry skin | 2 | 1/253 | 0/254 | 3.05 [0.13, 74.4] | Low | No inconsistency | No indirectness | No imprecision | Unlikely | High |
| Mucositis | 2 | 2/596 | 0/388 | 2.17 [0.23, 20.7] | Low | No inconsistency | No indirectness | No imprecision | Unlikely | High |
| Hand–foot-syndrome | 1 | 1/88 | 3/86 | 0.33 [0.03, 3.07] | Low | No inconsistency | No indirectness | No imprecision | Unlikely | High |
| Dermatitis | 1 | 0/88 | 0/86 | Not estimable | Low | No inconsistency | No indirectness | No imprecision | Unlikely | High |
| Arthralgia | 3 | 3/704 | 2/705 | 1.48 [0.25, 8.79] | Low | No inconsistency | No indirectness | No imprecision | Unlikely | High |
| Back pain | 3 | 6/704 | 3/705 | 1.85 [0.51, 6.72] | Low | No inconsistency | No indirectness | No imprecision | Unlikely | High |
| Myalgia | 3 | 7/704 | 5/705 | 1.35 [0.45, 4.03] | Low | No inconsistency | No indirectness | No imprecision | Unlikely | High |
| Myositis | 1 | 1/165 | 0/168 | 3.05 [0.13, 74.4] | Low | No inconsistency | No indirectness | No imprecision | Unlikely | High |
| Bone pain | 1 | 4/88 | 2/86 | 1.95 [0.37, 10.3] | Low | No inconsistency | No indirectness | No imprecision | Unlikely | High |
| Cough | 3 | 0/704 | 0/705 | Not estimable | Low | No inconsistency | No indirectness | No imprecision | Unlikely | High |
| Dyspnoea | 3 | 5/704 | 5/705 | 1.01 [0.29, 3.46] | Low | No inconsistency | No indirectness | No imprecision | Unlikely | High |
| Pneumonitis | 6 | 21/1770 | 4/1387 | 3.52 [1.34, 9.22] | Low | No inconsistency | No indirectness | No imprecision | Unlikely | High |
| Upper respiratory tract infection | 1 | 1/165 | 0/168 | 3.05 [0.13, 74.4] | Low | No inconsistency | No indirectness | No imprecision | Unlikely | High |
| Hypokalaemia | 1 | 11/451 | 4/451 | 2.75 [0.88, 8.57] | Low | No inconsistency | No indirectness | No imprecision | Unlikely | High |
| Hypertension | 2 | 14/616 | 23/619 | 0.62 [0.32, 1.18] | Low | No inconsistency | No indirectness | No imprecision | Unlikely | High |
| Hypotension | 1 | 0/88 | 0/86 | Not estimable | Low | No inconsistency | No indirectness | No imprecision | Unlikely | High |
| Decreased neutrophil count | 5 | 287/2035 | 181/1471 | 0.90 [0.76, 1.06] | Low | No inconsistency | No indirectness | No imprecision | Unlikely | High |
| Anemia | 6 | 269/2123 | 136/1557 | 1.17 [0.96, 1.42] | Low | No inconsistency | No indirectness | No imprecision | Unlikely | High |
| Neutropenia | 5 | 547/2054 | 319/1376 | 1.02 [0.91, 1.15] | Low | No inconsistency | No indirectness | No imprecision | Unlikely | High |
| Leucopenia | 2 | 44/253 | 38/254 | 1.14 [0.79, 1.66] | Low | No inconsistency | No indirectness | No imprecision | Unlikely | High |
| Febrile neutropenia | 3 | 28/322 | 30/435 | 1.28 [0.77, 2.12] | Low | No inconsistency | No indirectness | No imprecision | Unlikely | High |
| Thromboembolic event | 1 | 0/88 | 0/86 | Not estimable | Low | No inconsistency | No indirectness | No imprecision | Unlikely | High |
| Elevated alanine aminotransferase level | 5 | 96/2054 | 44/1376 | 1.39 [0.97, 1.99] | Low | No inconsistency | No indirectness | No imprecision | Unlikely | High |
| Hepatitis | 5 | 12/1204 | 1/1106 | 5.64 [1.52, 20.9] | Low | No inconsistency | No indirectness | No imprecision | Unlikely | High |
| Aspartate aminotransferase increased | 2 | 10/253 | 3/254 | 3.03 [0.91, 10.0] | Low | No inconsistency | No indirectness | No imprecision | Unlikely | High |
| Increased bilirubin | 1 | 0/88 | 0/86 | Not estimable | Low | No inconsistency | No indirectness | No imprecision | Unlikely | High |
| Hepatotoxicity | 1 | 0/88 | 0/86 | Not estimable | Low | No inconsistency | No indirectness | No imprecision | Unlikely | High |
| Adrenal insufficiency | 5 | 16/1900 | 0/1410 | 11.37 [2.22, 58.28] | Low | No inconsistency | No indirectness | No imprecision | Unlikely | High |
| Urinary tract infection | 1 | 4/451 | 3/451 | 1.33 [0.30, 5.92] | Low | No inconsistency | No indirectness | No imprecision | Unlikely | High |
| Increased creatinine | 1 | 0/88 | 0/86 | Not estimable | Low | No inconsistency | No indirectness | No imprecision | Unlikely | High |
| Hypothyroidism | 7 | 4/2554 | 0/1777 | 2.27 [0.38, 13.7] | Low | No inconsistency | No indirectness | No imprecision | Unlikely | High |
| Hypothyroidism | 7 | 6/2554 | 0/1777 | 3.82 [0.67, 21.6] | Low | No inconsistency | No indirectness | No imprecision | Unlikely | High |
| Diabetes | 2 | 4/596 | 2/388 | 1.02 [0.19, 5.53] | Low | No inconsistency | No indirectness | No imprecision | Unlikely | High |
| Hyperglycaemia | 1 | 0/88 | 0/86 | Not estimable | Low | No inconsistency | No indirectness | No imprecision | Unlikely | High |

**Abbreviations:** OS: overall survival; OSR: overall survival rate; PFS: progression-free survival; PFSR: progression-free survival rate; CI: confidence interval; AEs: adverse events.

^a^ Differences: hazard ratio (HR) for OS, PFS and complete response; risk ratios (RR) for OSR, PFSR, summary of adverse events, total adverse events and grade 3-5 adverse events.

^b^ Risk of bias assessed using the Jadad scale for randomized controlled trials.

^c^ Publication bias was explored through visual inspection of the funnel plots.
