# Supplementary figures and images for: Immune Checkpoint Inhibitors Combined With Chemotherapy Compared With Chemotherapy Alone for Triple-Negative Breast Cancer: A Systematic Review and Meta-Analysis
Source: Front Oncol. 2021 Dec 16;11:795650. doi: 10.3389/fonc.2021.795650 (PMC8716854; doi:10.3389/fonc.2021.795650)

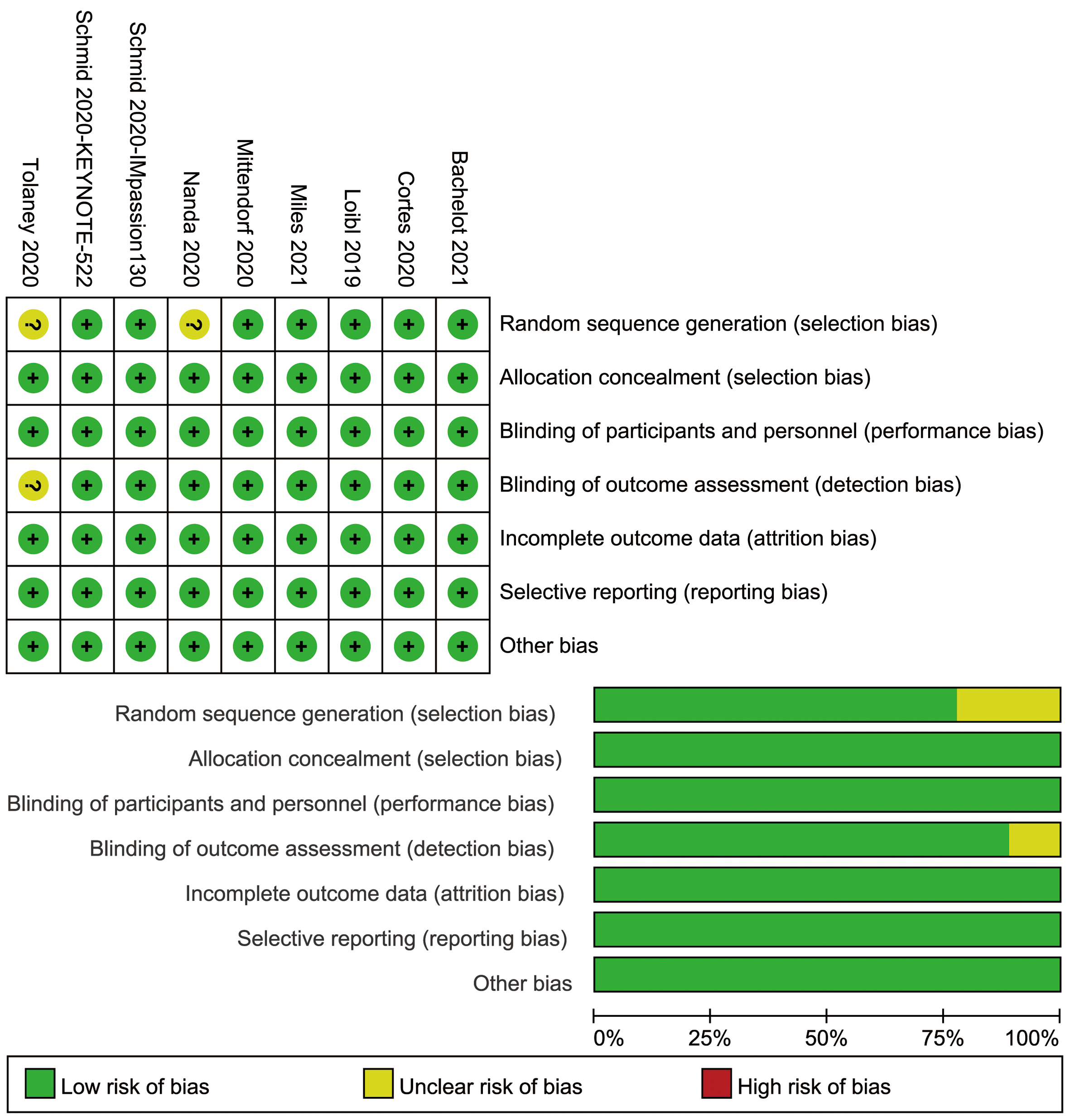

Supplement: Supplementary Figure 1 — Cochrane risk assessment associated with ICIs+Chemotherapy versus Chemotherapy. [file Image_1.tif]

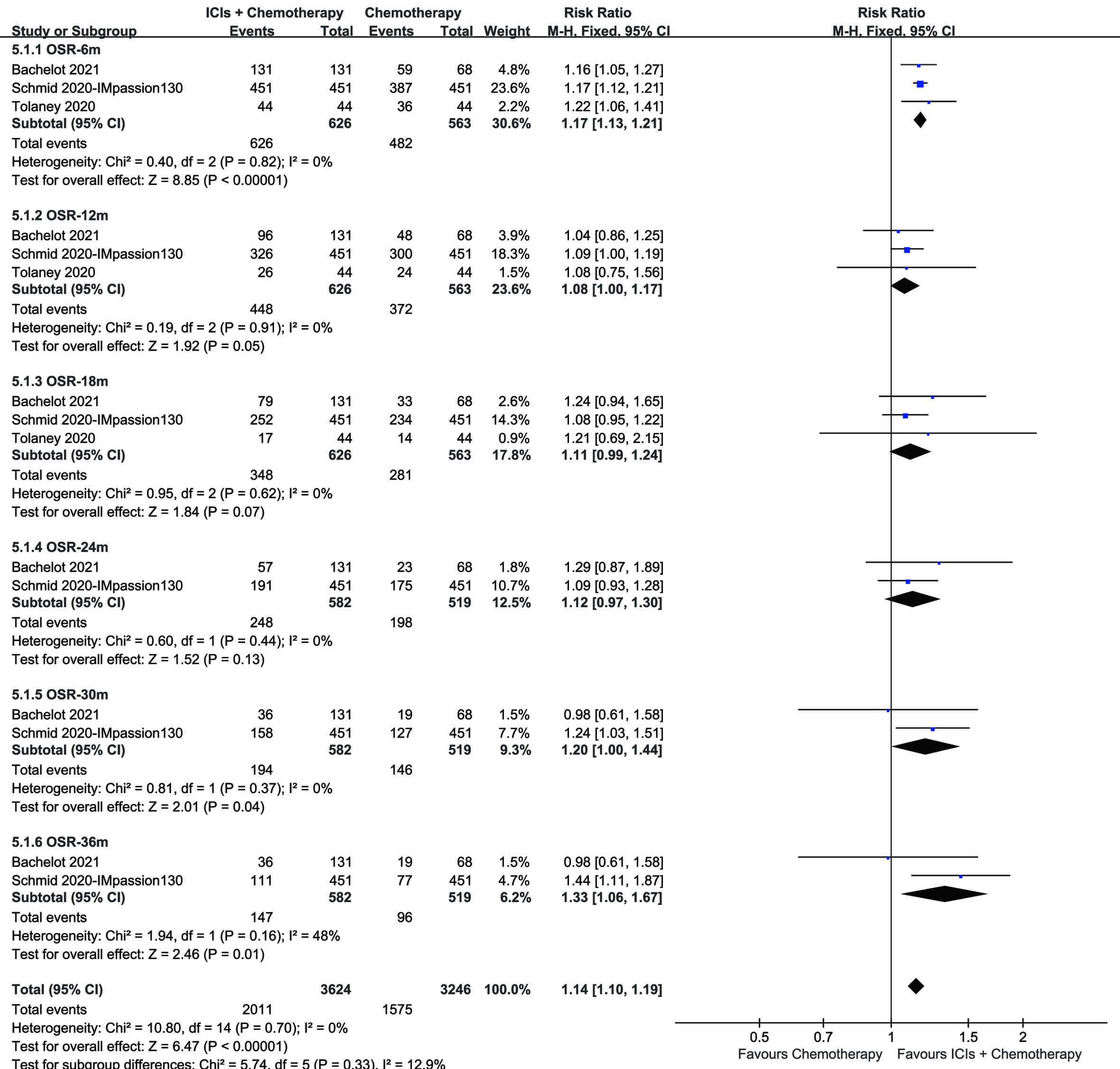

Supplement: Supplementary Figure 2 — Forest plots of OSR (6–36 months) associated with ICIs+Chemotherapy versus Chemotherapy according to survival time. [file Image_2.tif]

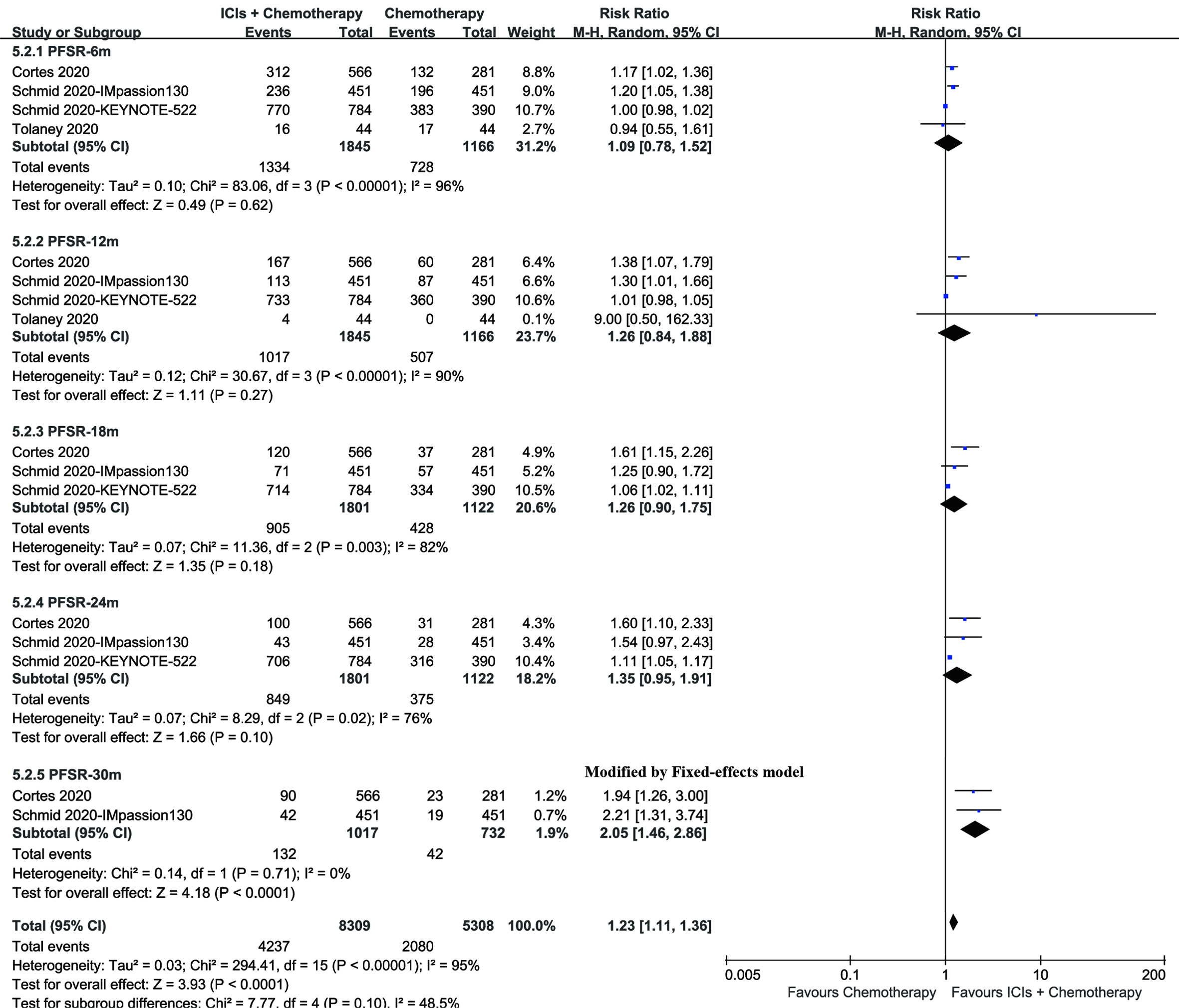

Supplement: Supplementary Figure 3 — Forest plots of PFSR (6–30 months) associated with ICIs+Chemotherapy versus Chemotherapy according to survival time. [file Image_3.tif]

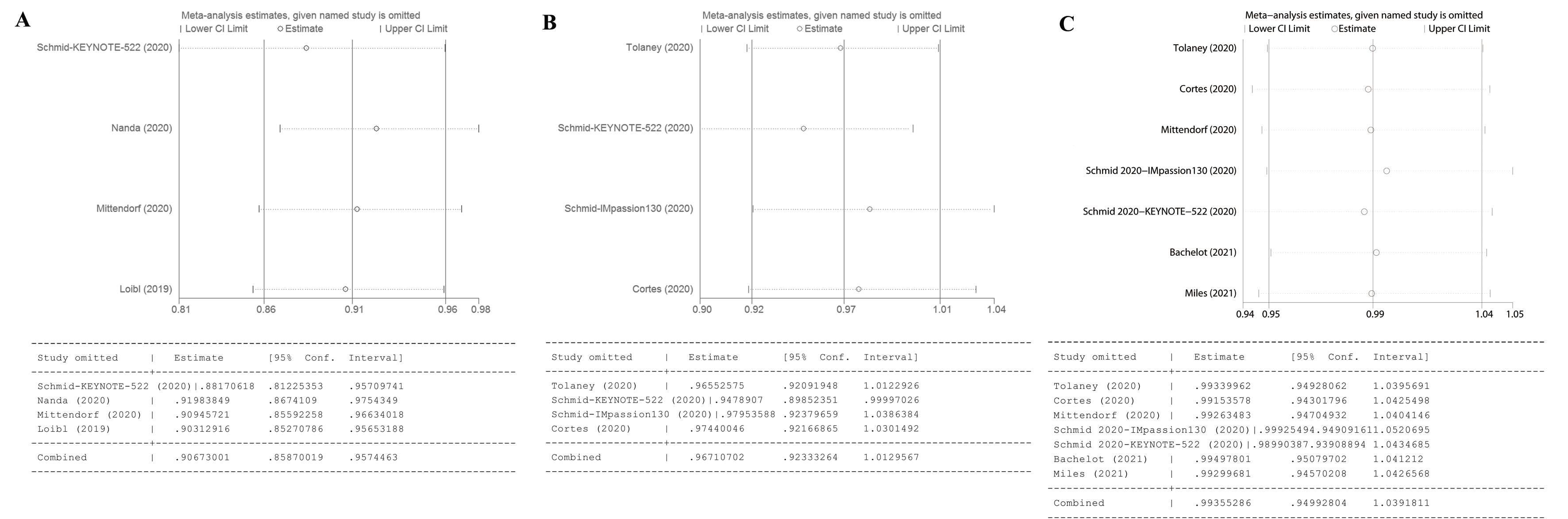

Supplement: Supplementary Figure 4 — Sensitivity analysis of complete response (A), PFSR (B), and total adverse events (C) associated with ICIs+Chemotherapy versus Chemotherapy. [file Image_4.tif]

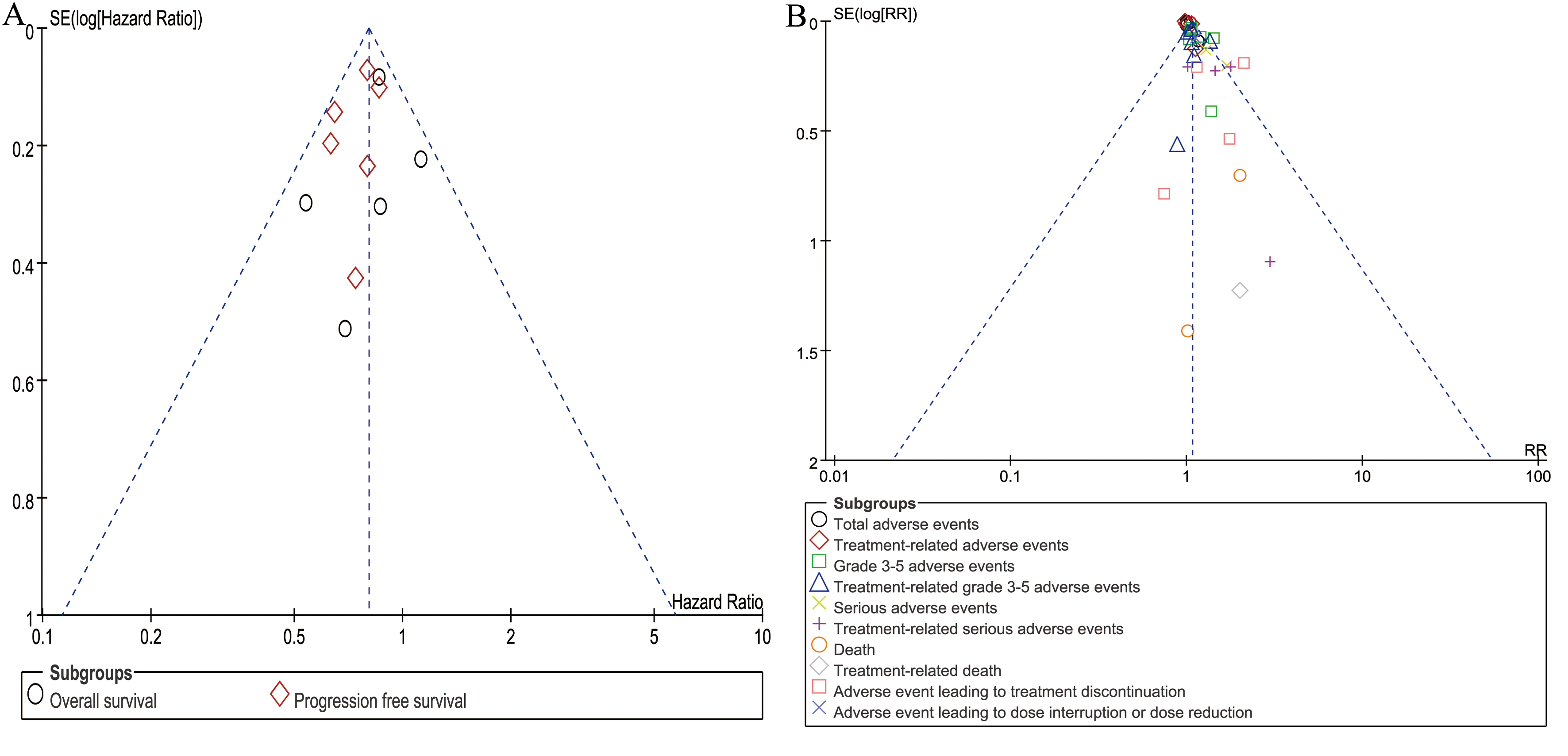

Supplement: Supplementary Figure 5 — Funnel plots of survival summary (A) and safety summary (B) associated with crizotinib versus alectinib. [file Image_5.tif]
